# Assessing chikungunya’s economic burden and impact on health-related quality of life: Two systematic literature reviews

**DOI:** 10.1371/journal.pntd.0012990

**Published:** 2025-05-05

**Authors:** Giorgia Tiozzo, Adrianne M. de Roo, Gabriel S. Gurgel do Amaral, Hinko Hofstra, Gerard T. Vondeling, Maarten J. Postma

**Affiliations:** 1 Asc Academics B.V., Groningen, The Netherlands; 2 Department of Health Sciences, University Medical Center Groningen, Groningen, The Netherlands; 3 Market Access and Value Evidence, Valneva, Vienna, Austria; 4 Department of Economics, Econometrics and Finance, Faculty of Economics and Business, University of Groningen, Groningen, The Netherlands; US Department of Homeland Security, UNITED STATES OF AMERICA

## Abstract

**Background:**

Chikungunya virus (CHIKV), transmitted through *Aedes* mosquitoes, has witnessed a global surge in distribution and outbreaks, posing a significant public health threat. This study conducted two systematic literature reviews (SLRs) to examine the socio-economic burden associated with chikungunya.

**Methods:**

Two SLRs were conducted employing the Preferred Reporting Items for Systematic Reviews and Meta-Analyses 2020 (PRISMA 2020) standards. The SLRs covered electronic databases, grey literature, and bibliographic searches. Inclusion criteria were guided by the PICOS (Population, Intervention, Comparator, Outcomes, Study type) framework.

**Findings:**

A total of 34 studies on cost and resource use and 37 studies on health-related quality of life (HRQoL) were included. Direct costs, driven by hospitalization, consultations, diagnostics, and drugs, were frequently reported, with significant variability across studies. Indirect costs, mainly attributed to absenteeism, constituted a substantial portion of the total economic burden. HRQoL studies consistently revealed high pain levels and diminished physical functioning among chikungunya patients, particularly in chronic cases, where these impacts persisted for months to years.

**Conclusions:**

This study highlights the significant economic and public health impact of chikungunya, driven by high treatment costs, productivity losses, and chronic disability. Despite the severity of the disease, limited awareness regarding the severity and economic burden, particularly in resource-limited regions, persists. Our findings underscore the need for targeted public health strategies, standardized management approaches, and increased research to better understand the socio-economic burden of the disease and inform effective interventions.

## Author summary

Chikungunya is a mosquito-borne illness that has rapidly spread across tropical and subtropical regions, presenting a substantial public health threat. The infection with the chikungunya virus (CHIKV) causes acute symptoms such as high fever and intense joint pain, but many patients suffer long-term effects, including chronic pain and disability, severely impacting their quality of life. Despite its socio-economic burden, chikungunya has received less attention than other mosquito-borne diseases like dengue and malaria. Our study highlights the financial strain on healthcare systems due to direct costs like hospitalizations, treatments, and medications, while indirect costs—primarily from lost productivity due to illness—further compound the economic impact. Furthermore, the chronic nature of chikungunya in many patients leads to lasting physical impairments and a profound reduction in quality of life, particularly in those suffering from chronic joint pain. By bringing attention to the substantial costs and quality of life reductions associated with chikungunya, our study calls for targeted strategies to reduce its socio-economic toll and improve outcomes for affected populations.

## Introduction

The chikungunya virus (CHIKV) is an arbovirus carried by mosquitos of the *Aedes* genus, particularly the *Aedes aegypti* and *Aedes albopictus*. CHIKV can be transmitted to humans by infected female mosquitoes through their bites, which can result in the onset of the disease in affected individuals. The most common symptoms that characterize chikungunya occur within four to seven days after mosquito contact and include high fever, skin rash, and polyarthralgia [1–4]. Death and severe health outcomes, however, tend to be rare and typically happen in older individuals [2–4]. Although most of the acute symptoms resolve within one to three weeks, around 43% of patients report persisting and debilitating polyarthralgia and polyarthritis [5–7]. These symptoms may become chronic, and the duration of their resolution can range from several months to several years. The long-term course of chikungunya has been shown to considerably impact the daily lives of patients and lower their health-related quality of life (HRQoL) [8,9]. Current measures against chikungunya primarily emphasize mosquito avoidance and symptom management [2,4]; however, the recent approval of the first prophylactic chikungunya vaccine by the US FDA underscores the growing necessity to investigate the economic implications of chikungunya to support healthcare decision-making [10].

Over the past few decades, the geographical spread of CHIKV has rapidly increased, as chikungunya cases have been reported in 115 countries [11]. Major outbreaks have occurred in tropical and subtropical regions such as Africa, Southeast Asia, the Indian subcontinent, the Pacific Region, and the majority of (sub)tropical regions of Central and South America. Until the present time, the affected regions continue to experience recurrent and notable CHIKV outbreaks. Notably, recent data from the Pan American Health Organization revealed a considerable upsurge in reported chikungunya cases within the region of the Americas, reaching more than 113 thousand cases, with 51 fatalities documented over a period of three months [12]. Furthermore, local autochthonous transmission and outbreaks have also occurred in Italy and France after the introduction of *Aedes albopictus* in Europe [13]. Climate change-related factors, such as global warming and increased precipitation, have altered the dynamics of mosquito distribution, thus raising the risk of CHIKV dissemination [14–16]. Moreover, human-related factors such as human mobility and globalization contribute to the virus importation to transmission-suitable areas [17]. The threat chikungunya can pose to public health in temperate regions has therefore increased as these circumstances have enabled CHIKV vectors to expand their habitat and survive in previously unsuitable areas [18]. The unprecedented increase in the spread of CHIKV over the past decade has led the WHO to shortlist chikungunya for inclusion in its Research and Development Blueprint for priority pathogens [19].

The combination of unpredictable and frequently rapid onset of chikungunya outbreaks—alongside the geographic expansion of areas suitable for CHIKV transmission—poses a significant threat to public health. This is especially concerning due to the potential for chronic and debilitating effects of the disease and the possible resulting economic burden on a larger scale. To mitigate this threat, organizations such as the WHO Global Arbovirus Initiative and Coalition for Epidemic Preparedness Innovations (CEPI) are actively supporting the development of strategic plans and vaccines against CHIKV and other re-emerging arboviruses. Despite these ongoing efforts of organizations like the WHO and CEPI, there is still a lack of representation of the full socio-economic disease burden. This underrepresentation is due to chikungunya being a neglected tropical disease, driven by underfunding and lack of research, low public awareness, economic and social challenges in endemic regions, historical neglect of diseases affecting marginalized populations, and prioritization of diseases with higher mortality rates. Therefore, the primary objective of this study is to provide a comprehensive understanding of the current health and economic burden attributable to chikungunya. To this purpose, two systematic literature reviews (SLRs) have been conducted to identify and summarize evidence on HRQoL outcomes and costs, and resource use associated with chikungunya. The study aims to inform policymakers regarding the establishment of public health initiatives against CHIKV as well as the implementation of potential novel interventions such as vaccines and antivirals.

## Methods

This study comprises two separate SLRs to gather evidence on HRQoL outcomes and cost and resource use associated with chikungunya. The Preferred Reporting Items for Systematic Reviews and Meta-Analyses 2020 (PRISMA 2020) standards were used to conduct these reviews. A protocol for this SLR was developed before the research began; however, this SLR was not registered in PROSPERO, and the protocol is not publicly available.

### Information sources and search strategy

The search was conducted on the electronic databases MEDLINE In-Process and Embase and MEDLINE since they represent standard evidence sources used in most health technology assessments. We built two different search queries for each electronic database, one for every subtopic of interest. The queries were built by combining free text items and Medical Subject Headings or Emtree subject headings. To create the search queries, we made use of the terms related to chikungunya, cost and resource use, and HRQoL. Exclusionary terms referring to comments, editorials, letters, and case reports were applied to rule out unwanted publication types. We also completed an additional grey literature search to identify the most recent evidence that might not have been detected by the electronic databases. Moreover, bibliographic searches of identified key systematic review and meta-analysis articles were performed to ensure that the initial searches captured all relevant studies. The complete lists of MEDLINE In-Process and Embase and MEDLINE search terms and grey literature data sources are provided in the ‘S1 File’.

### Eligibility criteria

The PICOS (Population, Intervention, Comparator, Outcomes, Study type) framework was used to determine the inclusion and exclusion criteria of the search. Two PICOS frameworks were created, one for each subtopic. These were then utilized to assess articles recognized by the search strings associated with their corresponding subtopic in order to identify the studies of interest. For all two subtopics, studies were included if the target population was affected by CHIKV or CHIKV in combination with other viruses. No limits were set for interventions or comparators. Outcomes and study design differed between the subtopics. For the costs and resource use evidence, studies were included if they reported any direct or indirect costs related to chikungunya as well as if they reported any frequency or magnitude of resource consumption. For the HRQoL evidence, studies were included if they reported any utility weights, disability-adjusted life years (DALYs), or other patient-reported outcome measurements (PROMs). For both searches, the exclusion criteria for the study design were in-vitro studies, preclinical studies, reviews, comments, letters, and editorials. Moreover, the searches were limited to studies in the English language. The exhaustive list of inclusion and exclusion criteria in accordance with the PICOS framework used for the three searches can be found in the ‘S1 File’.

### Study selection and data extraction

The retrieved studies were evaluated in two phases, during which they were juxtaposed against the eligibility criteria for the clinical search. In the first phase, two reviewers independently deduplicated and screened the titles and abstracts of the identified studies using Rayyan. In the second phase, the full texts of selected studies from this first phase were obtained and double-screened. In both phases, disagreements about the inclusion of a study were solved by reaching a consensus between the two researchers. Systematic reviews and meta-analyses were included in the first selection phase as a bibliographic search and then excluded during the second phase. To facilitate the data extraction of the included studies, a standardized evidence data extraction form was developed in DistillerSR. One reviewer performed data extraction while a second reviewer checked the data extraction of 10% of the included studies for quality control purposes. This process encompassed obtaining information on study design, patient characteristics, and outcomes of the included studies and was performed for full-text publications only. The two-phased screening processes and data extraction were performed separately for each of the two subtopics.

### Data synthesis and analysis

For each of the subtopics of interest, we created a summary of the main findings by means of building a narrative synthesis of the relevant information encompassed in the data extraction summary tables. No meta-analysis was performed due to the high heterogeneity of the study designs, patient population, and outcomes. To enable a comparison of costs across different years and currencies, all values were converted to 2023 USD. To enhance readability, the converted 2023 USD value is reported first, followed by the original value in its respective currency in square brackets.

## Results

**Fig 1:**
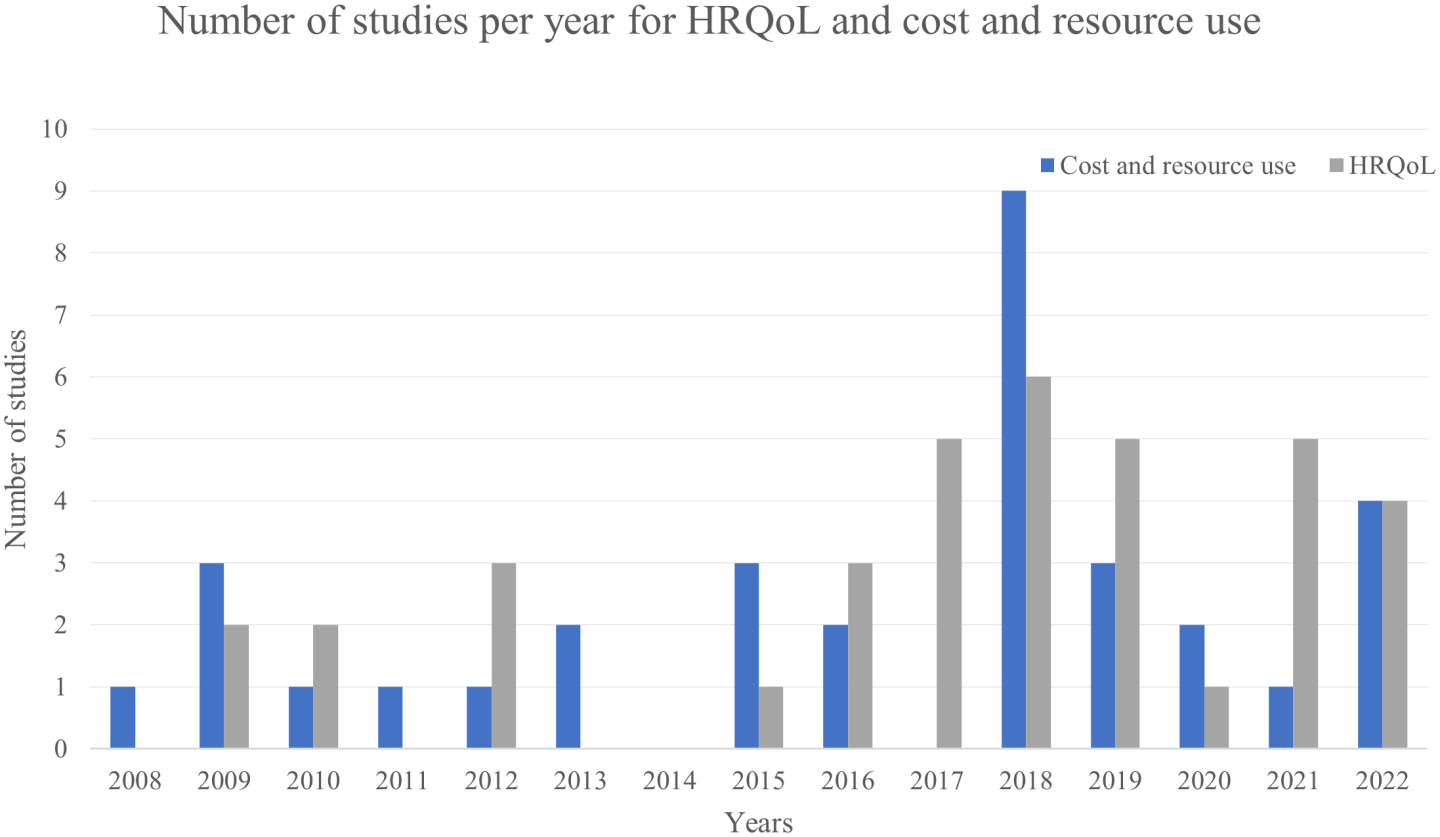
Number of studies per year on health-related quality of life (HRQoL) and cost and resource use. Most studies were published in 2018, with 9 studies on cost and resource use and 6 on HRQoL, while in 2014 no studies were published on HRQoL or cost and resource use.

The two searches for the SLRs were performed on 19 January 2023. After the duplicates had been removed, a total of 1,140 records were identified from the cost and resource use search and 260 from the HRQoL search. The details of the flow of studies included and excluded at each phase of the search are presented in the PRISMA flow diagrams in the ‘S1 File’. The final number of included studies was 34 for cost and resource use and 37 for HRQoL. The number of studies per year and per study type can be found in Fig 1.

### Costs and resource use

A total of 34 studies assessed the cost and resource use associated with chikungunya, including three economic evaluations, three real-world evidence studies, eight survey studies, and 20 observational studies. Studies were conducted across 11 locations, with the most common being Réunion Island, Colombia, and India. Of these, 10 articles focused on low- and middle-income countries (LMICs), 12 on upper-middle-income countries, and 12 on high-income countries. Most of the articles reported costs in USD.

Total outbreak costs varied widely, from $476,537.5 [R$1,301,520] in Brazil in 2016 [20] to $83,334,286.1 [€43,900,000.0] on Réunion Island in 2005 [21]. Estimated direct costs were reported in eleven studies, reporting a wide range of values. Inpatient care costs ranged from $9.9 [$6.5] per patient in India [22] to $21,857.5 [$16,982.0] in the U.S. Virgin Islands (USVI) [23], while outpatient care costs ranged from $39.8 [$30.9] per patient in Colombia [8] to $1,964.1 [$1,526.0] in the USVI [23]. Productivity losses per patient ranged from $13.5 [$8.9] in India [22] to $1,061.8 [$825.0] in the USVI [23]. An overview of all direct costs can be found in the ‘S1 File’.

The wide cost variations are largely attributable to the differing economic contexts and healthcare systems of the countries studied. In India, which is classified as a low-income country, the mean direct medical cost per patient was $48.4 [$32.0], with hospitalization costs averaging $9.9 [$6.5] per day, amounting to 50.8% of the country’s per capita healthcare expenditure (approximately $63) [24]. This includes a significant out-of-pocket expense in a country where more than 60% of healthcare expenses are privately financed [25]. In Colombia, an upper-middle-income country, median direct costs per patient were $410.4 [$318.9] for inpatient care and $39.8 [$30.9] for outpatient visits. This represents approximately 70.8% and 6.8% of Colombia’s per capita monthly income [26]. In high-income settings, such as the USVI, average outpatient costs were estimated at $1,964.1 [$1,526.0] and inpatient costs at $21,857.5 [$16,982.0] per patient. These costs are substantially higher than in LMICs, reflecting higher wages, resource availability, and social security coverage. However, even in high-income settings, chikungunya outbreaks imposed significant financial strain on public health budgets, with total outbreak costs estimated at $19.3–$42.8 million [$15–$33.3 million].

Most direct cost estimates typically included consultations, hospitalizations, drugs, and diagnostic use. In La Réunion’s 2005–2006 outbreak, consultation costs accounted for 47% of direct costs, hospitalization for 32%, and total hospitalization expenditures increased national healthcare costs by 25%, adding $23.5 million [€12.4 million] [21]. The total cost for all hospitalizations for chikungunya in La Réunion between 2005 and 2006 was $16.1 million [€8.5 million], with a mean of $3,796.5 [€2,000.0] per hospitalization and a mean duration of stay of 5 ± 7 days. Hospitalization rates ranged from 7.0% in Réunion Island [27] to 65.7% in French Guiana [28], while lengths of stay varied from 2.7 days in Colombia [29] and 17 days in Brazil [30]. Consultation rates ranged from 25% [23] to 93% [31], with the number of visits per patient ranging from 0.5 [23] to 9.5 [32] [S1 File]. Additionally, significant differences in resource use were identified between CHIKV-positive and CHIKV-negative individuals [31,32]. Data from La Réunion show that CHIKV-positive individuals have higher healthcare usage than CHIKV-negative individuals, with differences in consultations ranging from 9.5 per person for infected patients compared to 5.5 for uninfected in one study, and similar trends observed in another study. This increased healthcare consumption is particularly evident in visits to general practitioners and rehabilitation services, suggesting that CHIKV infection leads to significantly higher healthcare utilization overall.

Indirect cost estimates, reported in 10 studies, also showed substantial variation. The cost of absenteeism per patient for the studies in LMICs ranged from $13.4 to $43.2 [$8.9 to $29.4], with both values being reported in India [22,33]. In high-income regions, absenteeism-related costs were more pronounced. The total costs of absenteeism during the 2014–2015 outbreak in the USVI were estimated at $2.26 million [$1.76 million] [23]. The same study showed the cost per patient at two, six, and 12 months after disease onset, ranging from $917.7–$1,061.8 [$713.0–$825.0], $353.9–$409.3 [$275.0–$318.0], and $190.5–$221.4 [$148.0–$172.0], respectively. On Réunion Island, indirect costs due to sick leave contributed to 40% of the total costs during the 2005–2006 outbreak, with a national expenditure of $33 million [€17.4 million] [21] [S1 File]. A study in India estimated that chikungunya had higher indirect costs than dengue, with an average of $43.1 [Rs 2,550] for chikungunya and $32.6 [Rs 1,931] or dengue [34]. Absenteeism frequency reached 89% in a CHIKV-positive population in the first 2 months of illness [23], with the longest reported work absence lasting 35 days [35].

Overall, chikungunya’s economic burden varies by healthcare system, outbreak severity, and economic context. A detailed overview of the key findings of studies reporting resource use for CHIKV patients can be found in the ‘S1 File’.

### Health-related quality of life

The impact of chikungunya on HRQoL following CHIKV infection was evaluated in 37 studies, utilizing a wide range of methodologies within 30 different questionnaires. The visual analogue scale (VAS) was the most commonly adopted tool, employed in 19 studies to assess the level of pain experienced by patients (Table 1). In various studies, CHIKV patients consistently reported baseline VAS scores exceeding six, indicating a significant intensity of pain. The VAS ranges from 0 to 10, where 0 indicates no pain and 10 represents the worst possible pain. VAS scores above 6 are typically considered to indicate severe pain [36]. Only two studies reported VAS scores for the acute phase, both demonstrating a high pain burden in affected patients. The highest recorded mean VAS score of 8.3 was observed at baseline in a group of patients suffering from CHIKV-related fever and inflammatory polyarthritis within 6 weeks after infection [37]. VAS scores for chronic pain were more frequently reported, with mean VAS scores ranging from 5.27 to 8.02, indicating persistent pain or discomfort even months after the initial infection. Furthermore, the 36-Item Short Form Survey (SF-36) and the Health Assessment Questionnaire (HAQ) were employed in twelve and eight studies, respectively. Analysis of the SF-36 consistently revealed that CHIKV patients reported the worst scores in the dimensions of ‘bodily pain’ and ‘role functioning.’ Comparative assessments between recovered CHIKV patients and those still affected by CHIKV consistently indicated lower SF-36 scores in the latter group across all dimensions. In a study by Elsinga et al. [38], severely affected CHIKV patients reported significantly higher disability compared to mildly affected and recovered individuals[39]. Marimoutou et al. employed the MOS-SF36 questionnaire to evaluate HRQoL in three groups: healed CHIKV+ subjects, non-healed CHIKV+ subjects, and CHIKV- subjects. Healed CHIKV+ individuals scored significantly lower in all dimensions, except for physical functioning and general health, compared to CHIKV- subjects. Although they reported equivalent QoL, healed CHIKV+ patients experienced more rheumatic symptoms. Non-healed CHIKV+ subjects had lower scores than both other groups and lower scores than their pre-infection state, indicating higher levels of disability. These findings are consistently supported by additional questionnaires utilized in these studies. Importantly, a substantial number of the identified studies focused on investigating the outcomes of patients with persistent or chronic CHIKV infection, revealing significantly lower reported QoL, even two years after the onset of symptoms, when compared to healthy or recovered controls [39–41]. The level of pain (VAS) and disability (HAQ) in this patient group were comparable to individuals with rheumatoid arthritis [42–45]. A detailed overview of all studies and outcomes is provided in the ‘S1 File’.

**Table 1:** Overview of studies reporting HRQoL values using the Visual Analogue Scale (VAS) to measure pain in patients with chikungunya. The table summarizes key study details, including the VAS scores, which are categorized by acute and chronic chikungunya.

Author (year)	Study details • *Country* • *Population* • *Intervention*^1^	Acute disease VAS scores *(mean, ± SD or %)*	Chronic disease^2^ VAS scores *(mean, ± SD or %)*
Galate et al. (2016) [46]	• Mumbai, India • CHIKV patients with acute febrile illness (n = 200)	VAS score ≤5: 0% of patients VAS score 6–10: 100% of patients	
Padmakumar et al. (2009) [37]	• India • CHIKV infected patients (n = 120) within 6 weeks after CHIKV infection • Four different treatment groups.	VAS at baseline: Group 1: 8.30 ± 1.60 Group 2: 7.50 ± 1.6 Group 3: 7.90 ± 1.63 Group 4: 7.87 ± 1.46	
Amaral et al. (2019) [47]	• Brazil • CHIKV infected patients with arthritis >12 weeks (n = 35)		VAS (mean): 8.02 ± 1.82 VAS 7–10: 77% VAS 4–6: 20% VAS 1–3: 3%
Da Rocha et al. (2017a) *Abstract* [48]	• NR • CHIKV infected patients with neuropathic symptoms (n = 18)		VAS (pain): 4.4 ± 2.4 VAS (fatigue): 5.9 ± 2.9
De Andrade et al. (2010) [49]	• La Reunion, France • CHIKV infected patients (n = 106)		VAS (mean): 5.8 ± 2.1
De Oliveira et al. (2019) [50]	• Brazil • CHIKV patients with chronic musculoskeletal manifestations • Pilates		VAS at baseline: Pilates group: 6.7 ± 2.4 Control group: 7.4 ± 2.4
De Souza et al. (2021) [51]	• Brazil • CHIKV infected patients (women) with chronic arthralgia and VAS ≥ 4 (n = 58) • Transcranial direct current stimulation (tDCS)		VAS at baseline: Active-tDCS group: 6.86 ± 1.66 Sham-tDCS group: 6.07 ± 1.91
Doran et al. (2022) [40]	• Curaçao, Netherlands • CHIKV infected patients, either recovered or still affected after 60 months of disease onset		Recovered at 60 months after disease onset (n = 107): - VAS score 0: 73.8% - VAS score mild (1–3): 12.1% - VAS score moderate (4–6): 5.5% - VAS score severe (7–10): 8.4% Affected at 60 months after disease onset (n = 62) - VAS score 0: 73.8% - VAS score mild (1–3): 12.1% - VAS score moderate (4–6): 5.5% - VAS score severe (7–10): 8.4%
Frye et al. (2018) *Abstract* [52]	• Haiti • CHIKV patients with symptoms for at least 6 months (n = 67) • Homeopathic medication		VAS at baseline: 6.87 ± 5.15
Jain et al. (2017) [53]	• India • CHIKV infected patients 12 weeks post onset of fever (n = 130)		VAS (median): 6.05 VAS score 0–5: 34.6% of patients VAS score 6–10: 65.4% of patients
Kamal et al. (2021) [54]	• Bangladesh • CHIKV infected patients with VAS score ≥4 (n = 21) • Amitriptyline or duloxetine after 1, 2, 3 and 4 weeks		VAS at baseline: Amitriptyline group: 5.65 ± 0.94 Duloxetine group: 5.45 ± 0.78
Marques et al. (2016) *Abstract* [55]	• Brazil • CHIKV infected patients with rheumatic disease prior to CHIKV infection (n = 33)		VAS (pain): 6.36 ± 2.52 VAS (stiffness): 7.44 ± 2.11
Martin et al. (2019) *Abstract* [56]	• Colombia • 548 patients with clinical suspicion of CHIKV infection, with 295 positive testing for CHIKV		EQ-5D-VAS — VAS: 75.56 ± 21.18
Neumann et al. (2021) [57]	• Brazil • Chronic CHIKV (n = 31) • Resistance exercise		VAS at baseline: Resistance exercise group: 6.43 ± 2.4 Control group: 6.12 ± 2.0
Porangaba et al. (2019) *Abstract* [45]	• NR • CHIKV infected patients (n = 69) with persistent musculoskeletal symptoms after 4 weeks		Subacute phase (3–12 weeks) VAS 6.84 ± 1.9 Chronic phase (12–24 weeks) VAS 5.27 ± 2.22 Chronic phase (+24 weeks) VAS 5.64 ± 2.3
Ravindran et al. (2017) [58]	• India • Chronic persistent arthritis for >1 year after CHIKV infection • Combination therapy with methotrexate, sulfasalazine, and hydroxychloroquine or monotherapy with hydroxychloroquine.		VAS at baseline (VAS-100mm) Combination therapy: 64.32 ± 1.75 Monotherapy: 62.57 ± 2.05
Watson et al. (2019) *Abstract* [42]	• Colombia • 81 patients with chronic chikungunya		VAS-100mm: 65 ± 20
Watson et al. (2021) [43]	• Brazil • 40 patients with chronic chikungunya arthritis		VAS-100mm: 73 (50–82)

CHIKV: Chikungunya Virus; NR: not reported; SD: standard deviation; tDCS: transcranial direct current stimulation; VAS: Visual Analog Scale.

Notes:

^1^From interventional studies, only the baseline VAS score is reported. One abstract reported no baseline values, therefore this study by Alam et al. (2018) is excluded from this table [59].

^2^Chronic disease is defined as any symptoms persisting 3 months after disease onset.

## Discussion

Two SLRs were carried out to identify and summarize evidence on HRQoL outcomes as well as costs and resource use associated with chikungunya. Over the past years, CHIKV has experienced a rapid increase in terms of distribution and outbreak frequency, becoming a global health concern. Understanding its economic burden and impact on the HRQoL of patients is crucial to inform public health interventions and resource allocation.

Our findings on costs outcomes for chikungunya align with previous studies [60]; however, our broader scope allowed us to identify a considerably larger number of studies, offering a more extensive analysis of the economic burden associated with chikungunya. Direct costs, reported more frequently than indirect costs, were driven by hospitalization, consultations, diagnostics, and drugs, with inpatient costs prevailing during the acute phase and outpatient costs during the chronic phase. Indirect costs were mostly associated with productivity losses due to absenteeism and contributed significantly to the total costs of chikungunya. The costs and resource use varied considerably across studies, primarily due to the differences in income of the affected countries as well as the high heterogeneity in study settings, population, healthcare system, measurement tools, outbreak intensities, and differences across studies in reporting the outcomes.

Compared to other arboviral diseases such as dengue, chikungunya has lower total global costs. However, prolonged post-acute symptoms and higher chronicity rates related to chikungunya may lead to greater indirect costs over time [61,62]. Globally, annual costs for dengue are estimated at $11.6 billion [$8.9 billion], while annual costs associated with chikungunya are estimated at $5.85 billion [$5.0 billion] [61,63]. Direct cost per case for dengue ranges from $73.2 to $1,498.9 [$56.0 to $1,146.0], depending on the region [61], whereas, for chikungunya, direct costs per case range from $9.9 [$6.5] in India to $21,857.5 [$16,982.0] in the USVI. Higher case numbers worldwide, combined with considerable hospitalization expenditures, cause the substantial total economic burden of dengue. Nevertheless, per-person indirect costs associated with chikungunya are often demonstrated to be higher, largely due to its chronic symptoms and prolonged productivity losses [60,63,64]. This significant contribution of indirect costs underscores the broader socio-economic impact of the disease and highlights the importance of long-term management strategies in affected regions.

Chikungunya disproportionately affects LMICs, where healthcare infrastructure and resources are often limited. Despite this, our study found that only 10 of 34 economic evaluations focused on these regions, revealing a critical data gap. While direct costs per patient in LMICs are generally lower (e.g., $3.3 [$2.2] per patient in a health centre in LMIC versus $21,857.5 [$16,982.0] per inpatient visit in high-income countries), they represent a much larger relative economic burden, straining already limited healthcare budgets. The economic burden in LMIC regions is amplified by high out-of-pocket healthcare expenditures, as limited insurance coverage forces individuals and families to bear treatment costs. These expenses can represent a significant portion of household income, sometimes leading to catastrophic health expenditures [65]. Additionally, the economic reliance on daily wages means that disease-related absenteeism can have severe financial consequences. Unlike in high-income settings where paid sick leave is common, many individuals in LMICs—especially those in informal employment—experience income loss when unable to work [66]. Caregivers also face economic strain as they forgo work to care for sick family members.

Underreporting and misdiagnosis, often due to symptom overlap with dengue, likely lead to an underestimation of chikungunya’s true burden. This is especially concerning given chikungunya’s high symptomatic rate (74.9%) and chronicity rate (43.89% at three months post-onset) [67], which contribute to prolonged absenteeism and productivity losses. Barriers to healthcare access, including geographic inaccessibility, financial constraints, and workforce shortages, contribute to delayed diagnoses and an underestimation of the disease’s prevalence and costs [68]. Limited healthcare access may also increase the risk of complications, reinforcing the need for standardized disease management [69].

Beyond healthcare costs, chikungunya outbreaks in LMICs disrupt social cohesion, education, and economic stability, placing additional strain on public resources [70]. These challenges highlight the value of targeted interventions to mitigate the broader health and economic impacts of the disease, particularly in vulnerable regions [60].

The SLR on HRQoL outcomes shows that chikungunya patients consistently reported high levels of pain and low scores in physical functioning across health questionnaires. Particularly, patients with chronic and persistent chikungunya appeared to report low scores in physical functioning months, and even years, after disease onset, presenting similar QoL outcomes as rheumatoid arthritis patients. This outcome highlights the profound impact on QoL during the chronic phase of chikungunya, emphasizing the substantial disability associated with the long-term impact of the disease. In the absence of specific disability weight (DW) data for chikungunya, four studies resorted to using DW values established for osteoarthritis and rheumatoid arthritis to estimate the DW range (0.16–0.23) for chronic CHIKV cases [22,71–73]. Additionally, in the context of acute chikungunya, DW values for dengue were employed [22,71,73]. Notably, none of the studies included in the review reported utilities, which are measures of the value individuals place on different health states. Health utilities are commonly used in cost-effectiveness analyses to assess the QoL associated with diseases and treatments, highlighting the lack of awareness and data concerning this disease. The QoL studies presented a wide range of preference-based methods used to measure HRQoL, decreasing the comparability of outcomes. In conclusion, the high incidence of morbidity and the persistent long-term sequelae in combination with the poor QoL reported by infected patients highlight the high burden that the disease can cause. Future research should focus on determining utility weights for comprehensive quality-adjusted life year assessments as well as perform more prospective studies to avoid recall bias and obtain more accurate estimates of the actual disease burden. While previous research focused primarily on long-term sequelae and included five studies on quality of life, our study takes a complementary approach by evaluating outcomes from 37 studies, including both acute and chronic chikungunya. This broader scope offers an additional perspective on the economic impact and health-related quality of life associated with the disease [74].

Among the included studies, only one health economic model investigated and compared the cost and health outcomes of several interventions to prevent and control the spread of CHIKV and dengue virus (DENV) [72]. The study compared the health benefits and cost-effectiveness of a hypothetical CHIKV vaccine, a hypothetical DENV vaccine, mosquito control via insecticide, and the combination of these interventions, using mono– and multi-disease models. In the model focused exclusively on chikungunya cases, the highest cost-effectiveness, as measured by the net monetary benefit (NMB), was achieved by using the CHIKV vaccine alone. In the multi-disease model, the highest NMB was obtained by using only insecticides and the CHIKV vaccine. While vector control measures are effective interventions, this study highlights the need for additional interventions, such as vaccines, which have shown a potential for cost-effectiveness, especially in the long term. Given the absence of economic modelling studies, drawing comprehensive conclusions regarding the cost-effectiveness of chikungunya interventions becomes challenging. One reason for the previously scarce literature on economic modelling studies in this context may have been the lack of approved interventions to prevent or cure chikungunya, a situation that has only recently changed with the approval of the first chikungunya vaccine. Consequently, more studies in this modelling field—which could use data from these two SLRs—are needed to better determine cost-effectiveness measures aimed at controlling the spread or treatment of chikungunya.

The limited number of articles identified in our study highlights the lack of awareness about chikungunya, despite its significant potential to affect local economies and the quality of life of individuals. This low awareness may stem from chikungunya often being misdiagnosed as other, more familiar diseases like malaria and dengue, particularly in high-risk areas where these diseases are prevalent. Symptoms overlap with these infectious diseases, and coinfections can occur, complicating diagnosis and reporting. Improving the recognition of chikungunya, both clinically and socially, is crucial to understanding the full impact of the disease and ensuring the adoption of effective preventive measures. Additionally, the high heterogeneity in outcomes across studies, even under existing WHO and other health authority guidelines, reflects the complexity of chikungunya’s global impact. This heterogeneity is attributable to variations in study settings, populations, and measurement tools, as well as disease management practices among countries (such as under- and over- reporting, testing policies, and medication choices). Future efforts should prioritize research that fully evaluates the economic and health impacts of chikungunya at both national and individual levels. Moreover, developing a standardized approach to chikungunya management and prevention is essential for reducing the socio-economic burden in affected regions.

This study has several limitations, both in terms of methodology and the evidence available at the time of review. First, the data identified were limited, which may be attributed to the overall lack of awareness about chikungunya among both the general population and healthcare professionals. Additionally, the exclusion of non-English language publications may have contributed to the scarcity of identified studies; however, further searches in PubMed, Embase, and SciELO for non-English articles resulted in only a small number of potentially relevant studies. Furthermore, the English-language studies included in our review are broad, robust, and encompass the regions most affected by chikungunya, thereby providing a comprehensive overview of the disease’s impact. Nevertheless, while our focus on English-language publications was guided by practical considerations, future research should aim to include a wider range of languages to further enhance the global understanding of chikungunya. Another limitation of this study was the high level of heterogeneity across studies. The high variety of studied populations, study design, and settings—as well as healthcare systems with regard to costs—made comparing the outcomes and pooling the data together difficult. Consequently, conducting a meta-analysis was deemed infeasible as the outcomes presented limited comparability. To enable the execution of a meta-analysis, the review would require a more focused scope by imposing restrictions on study design, country selection, or differentiating between distinct phases of the disease, thereby enhancing the comparability of the studies. Additionally, there is a possibility that primary data may not have been consistently reported across all included studies, which could introduce some degree of information bias.

## Conclusion

Chikungunya imposes a significant economic burden, particularly in low- and middle-income countries where limited healthcare resources and high out-of-pocket costs exacerbate the impact. Direct costs, such as hospitalization and outpatient care, are compounded by substantial indirect costs due to long-term productivity losses, especially in the chronic phase. While chikungunya has lower total global costs compared to dengue, its potential long-term symptoms contribute to a greater long-term socio-economic burden. The lack of standardized disease management and utility weights for HRQoL further complicates burden assessments. Our findings highlight the potential for chikungunya to impose a substantial socio-economic burden, especially in the context of large outbreaks, where morbidity rates and the risk of chronicity are increased. Future research should prioritize improved surveillance, cost-effectiveness modelling of vaccination strategies, and the development of standardized interventions to mitigate the disease’s impact globally.

## Supporting information

S1 File Containing the search strategy and PICOs of the studies included in the SLR, PRISMA flow diagrams, summary of study characteristics and key findings, and quality assessment of included studies. (PDF)

S2 File Checklist comprising 27 items addressing the introduction, methods, results and discussion sections of a systematic review report. (PDF)
