# Supplementary material for: Assessing chikungunya’s economic burden and impact on health-related quality of life: Two systematic literature reviews
Source: PLoS Negl Trop Dis. 2025 May 5;19(5):e0012990. doi: 10.1371/journal.pntd.0012990 (PMC12074603; doi:10.1371/journal.pntd.0012990)
Supplement: S1 File — (PDF) [file pntd.0012990.s001.pdf]

Assessing Chikungunya's Economic Burden and Impact on Health-Related Quality of Life: Two Systematic Literature Reviews

Giorgia Tiozzo[1,2*], Adrianne de Roo[3], Gabriel S Gurgel do Amaral[1], Hinko Hofstra[1], Gerard T Vondeling[3], Prof Maarten J Postma Ph.D.[2,4]

1. Asc Academics B.V., Groningen, the Netherlands
2. Department of Health Sciences, University Medical Center Groningen, Groningen, the Netherlands
3. Valneva, Market Access & Value Evidence, Vienna, Austria
4. Department of Economics, Econometrics and Finance, University of Groningen, Faculty of Economics & Business, the Netherlands

* Correspondence: giorgia.tiozzo@ascacademics.com;

Supplementary material

Methods

1. Study selection criteria

Table 1. PICOS Criteria for the inclusion and exclusion of the cost and resource use.

Category	Inclusion criteria	Exclusion criteria
Population (P)	Chikungunya patients or articles focusing on Chikungunya.	None
Interventions (I)	Any	None
Comparators (C)	Any	None
Outcomes (O) (tentative list, not exhaustive)	Direct costs by health state, including the following categories:<br>Medication costs<br>Outpatient visit costs<br>Hospitalization costs<br>Laboratory costs<br>Diagnostic costs<br>Physician costs<br>Indirect or other costs of interest by health state, including the following:<br>Costs for assistance/assisted living<br>Productivity loss of patient (wages lost from absence)<br>Out-of-pocket expenses<br>Travel costs for patient<br>Resource-use estimates by health state (e.g., number of hospitalizations and length of stay, drug utilization, physician visits)	None
Study design (S)	Cost studies<br>Resource use studies<br>Economic evaluations reporting costs or resource use<br>Budget impact	*In vitro* studies<br>Preclinical studies<br>Reviews, comments, letters, and editorials
Language	English	None
Time limit	No restriction	None
Country	No restriction	None

Table 2. PICOS Criteria for the inclusion and exclusion of health-related quality of life (HRQoL).

Category	Inclusion criteria	Exclusion criteria
Population (P)	Chikungunya patients or articles focusing on Chikungunya.	None

Interventions (I)	Any	None
Comparators (C)	Any	None
Outcomes (O)<br><br>(tentative list, not exhaustive)	HRQoL outcomes health state (e.g., EuroQol 5 dimensions [EQ-5D], Health Utilities Index (HUI), Short-Form 6-D (SF-6D) [b], DALYs, other patient-reported outcomes (PRO)	None
Study design (S)	Studies reporting utility/disutility data – QoL<br>Economic modelling studies<br>Studies reporting utility values (e.g. EQ-5D, SF-36, etc.)<br>Studies reporting mapped utility values<br>Studies reporting elicited utility data from the general population	*In vitro* studies<br>Preclinical studies<br>Reviews, comments, letters, and editorials<br>Case reports, case series<br>Clinical studies reporting only efficacy and safety data
Language	English language	None
Time limit	No restriction	None
Country	No restriction	None
Abbreviations: EQ-5D, EuroQol 5 dimensions; HUI, Health utilities index; PRO, patient-reported outcome, SF-6D, Short-Form 6D; QoL, Quality of life.		

2. Data sources and search strategy

2.1. Electronic databases

The following electronic databases were searched (i.e., standard evidence sources used in UK HTA assessments) to identify the relevant literature:

- MEDLINE and Embase (Embase.com)
- MEDLINE In-Process (PubMed.gov)

Based on findings from a study by Bramer et al. which highlights that Embase and MEDLINE together achieve a recall of at least 90% in most reviews, we focused on these databases for their broader coverage and high recall rates (1). The inclusion of databases like LILACS and SciELO was deemed unnecessary, as the study found they did not contribute unique references, further supporting our approach.

2.2. Grey literature search

A grey literature search was conducted to help identify the most recent abstracts, posters, and podium presentations that may not have been indexed in the medical literature databases. These searches were limited to the last four years (January 2019– January 2023) to capture the most recent unpublished or ongoing trials. The search covered the following conferences:

- American Society of Tropical Medicine & Hygiene (ASTMH) Annual Meeting

- International Conference on Clinical Microbiology and Infectious Disease Epidemiology (ICCMIDE)
- Conference of the International Society of Travel Medicine (CISTM)
- European Congress of Clinical Microbiology & Infectious Diseases (ECCMID)
- Northern European Conference on Travel Medicine (NECTM)
- International Conference on Tropical Medicine and Infectious Diseases (ICTROMI)
- International Society for Pharmacoeconomics and Outcomes Research (ISPOR): ISPOR Europe, ISPOR-FDA, ISPOR Asia Pacific, ISPOR Latin America, ISPOR Warsaw, ISPOR Dubai

2.3. Search terms

2.3.1. HRQoL search strategy

Table 3. HRQoL Embase pilot search strategy (Jan 19, 2023).

String No.	Query	Hits
**1**	‘chikungunya’/exp OR ‘chikungunya’:ab,ti	10,348
**2**	'european quality of life 5 dimensions questionnaire'/exp OR 'short form 36'/exp OR 'patient preference'/exp OR 'visual analog scale'/exp OR 'quality of life'/exp OR utilit*:ab,ti OR disutilit*:ab,ti OR 'sf 6':ab,ti OR sf6:ab,ti OR 'short form 6':ab,ti OR 'shortform 6':ab,ti OR 'sf six':ab,ti OR 'sfsix':ab,ti OR 'shortform six':ab,ti OR 'short form six':ab,ti OR 'sf 36':ab,ti OR sf36:ab,ti OR 'short form 36':ab,ti OR 'shortform 36':ab,ti OR 'sf thirtysix':ab,ti OR 'sfthirtysix':ab,ti OR 'shortform thirtysix':ab,ti OR 'short form thirtysix':ab,ti OR euroqol:ab,ti OR 'euro qol':ab,ti OR eq5d:ab,ti OR 'eq 5d':ab,ti OR 'health utilities index':ab,ti OR hui:ab,ti OR hui1:ab,ti OR hui2:ab,ti OR hui3:ab,ti OR ((standard NEXT/1 gamble*):ab,ti) OR 'quality of life*':ab,ti OR 'time trade off':ab,ti OR 'time tradeoff':ab,ti OR tto:ab,ti OR ‘visual analog scale’:ab,ti OR ‘patient preference’:ab,ti OR 'european quality of life 5 dimensions questionnaire':ab,ti OR QALY:ab,ti OR ‘quality-adjusted life year’:ab,ti OR DALY:ab,ti OR ‘disability-adjusted life year’:ab,ti	1,222,586
**3**	'case study'/it OR 'case report'/it OR 'abstract report'/it OR editorial/it OR ‘veterinary clinical trial’/it OR letter/it OR note/it	2,890,512
**4**	#1 AND #2	285
**5**	#4 NOT #3	260

Table 4. HRQoL PubMed pilot search strategy (Jan 19, 2023).

String No.	Query	Hits
**1**	“Chikungunya fever”[Mesh] OR “chikungunya”[tiab]	7,007
**2**	“Patient Health Questionnaire”[MeSH] OR “patient preference”[MeSH] OR “quality of life”[MeSH] OR “visual analog scale”[MeSH] OR utilit*[tiab] OR disutilit*[tiab] OR “sf 6”[tiab] OR sf6[tiab] OR “short form 6”[tiab] OR “sf six”[tiab] OR “sfsix”[tiab] OR “short form six”[tiab] OR “sf 36”[tiab] OR sf36[tiab] OR “short form 36”[tiab] OR “shortform 36”[tiab] OR euroqol[tiab] OR “euro qol”[tiab] OR eq5d[tiab] OR “eq 5d”[tiab] OR “health utilities index”[tiab] OR hui[tiab] OR hui1[tiab] OR hui2[tiab] OR hui3[tiab] OR (standard[tiab] AND gamble*[tiab]) OR “quality of life*”[tiab] OR “time trade off”[tiab] OR “time tradeoff”[tiab] OR tto[tiab] OR “visual analog scale”[tiab] OR “patient preference”[tiab]	735,673
**3**	“case reports”[pt] OR editorial[pt] OR letter[pt] OR comment[pt] OR “clinical trial, veterinary”[pt]	4,231,728
**4**	#1 AND #2	132
**5**	#4 NOT #3	124

2.3.2. Cost and resource use search strategy

Table 5. Costs and resource use Embase pilot search strategy (Jan 19, 2023).

String No.	Query	Hits
**1**	‘chikungunya’/exp OR ‘chikungunya’:ab,ti	10,348
**2**	'cost control'/exp OR 'cost of illness'/exp OR 'health care cost'/exp OR 'health care utilization'/exp OR 'resource management'/exp OR 'length of stay'/exp OR 'economic aspect'/exp OR ((('health care' OR healthcare) NEXT/1 (cost OR costs OR utilization OR utilisation)):ab,ti) OR ((resource NEXT/2 (allocat* OR utilization OR utilisation OR use OR management)):ab,ti) OR price*:ab,ti OR pricing:ab,ti OR economic*:ab,ti OR cost:ab,ti OR costs:ab,ti OR ‘cost control’:ab,ti OR ‘cost of illness’:ab,ti OR ‘length of stay’:ab,ti OR ‘economic aspect’:ab,ti	33,314,962
**3**	'case study'/it OR 'case report'/it OR 'abstract report'/it OR editorial/it OR ‘veterinary clinical trial’/it OR letter/it OR note/it	2,890,512
**4**	#1 AND #2	1,347
**5**	#4 NOT #3	1,240

Table 6. Costs and resource use PubMed pilot search strategy (Jan 19, 2023).

String No.	Query	Hits
**1**	“Chikungunya fever”[Mesh] OR “chikungunya”[tiab]	7,007
**2**	“cost control”[MeSH] OR “cost of illness”[MeSH] OR “health care costs”[MeSH] OR “health care economics and organizations”[MeSH] OR “Patient Acceptance of Health Care”[MeSH] OR “health resources”[MeSH] OR “length of stay”[MeSH] OR (((“Delivery of Health Care”[tiab]) AND (cost[tiab] OR costs[tiab] OR utilization[tiab] OR utilization[tiab]))) OR (resource[tiab] AND (allocat*[tiab] OR utilization[tiab] OR utilization[tiab] OR use[tiab] OR management[tiab])) OR price*[tiab] OR pricing[tiab] OR economic*[tiab] OR cost[tiab] OR costs[tiab] OR “cost control”[tiab] OR “cost of illness”[tiab] OR “length of stay”[tiab] OR “economic aspect”[tiab]	2,665,861
**3**	“case reports”[pt] OR editorial[pt] OR letter[pt] OR comment[pt] OR “clinical trial, veterinary”[pt]	4,231,728
**4**	#1 AND #2	560
**5**	#4 NOT #3	543

2.4. Study selection and data collection

All retrieved studies were assessed against the eligibility criteria for the clinical search. The study selection process was performed in the following two phases:

- Primary (Level 1) screening: titles and abstracts of studies identified from the electronic databases and Internet searches were double screened by two independent researchers to determine eligibility according to the inclusion and exclusion criteria described in Table 1 in the main text. In case of disagreement about study relevance, a consensus was reached through a discussion between the two researchers.
- Secondary (Level 2) screening: full texts of studies selected at Level 1 were obtained and double-screened by two independent researchers to determine eligibility according to the inclusion and exclusion criteria. In case of disagreement, a consensus was reached through a discussion between the two researchers.

2.5. Risk of bias

Two independent reviewers performed a quality assessment for all included studies. The quality of the cost and resource use studies was performed using the British Medical Journal checklist, as recommended by ISPOR Good Practices Task Force Report (2,3). As there is no definite checklist to specifically assess QoL, previous studies have used modified or own-developed checklists in their assessments (4–6). Therefore, the quality assessment of HRQoL studies in our SLR for those studies was performed utilizing the methodology from a previous SLR that developed an adapted checklist based on other published HRQoL studies (7). The checklists with the quality assessment of cost and resource use and HRQoL studies can be found in Table 10 and Table 11. (7)

Results

1. PRISMA flow diagrams

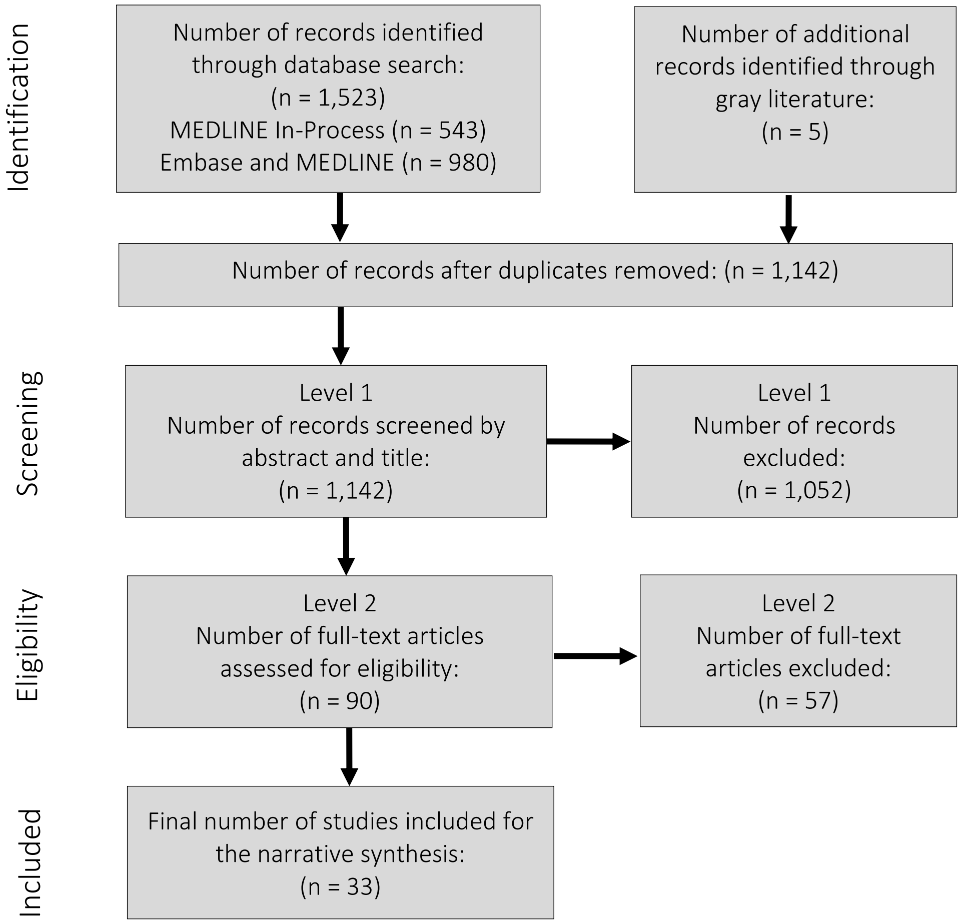

Figure 1. PRISMA flow diagram for the cost and resource use SLR

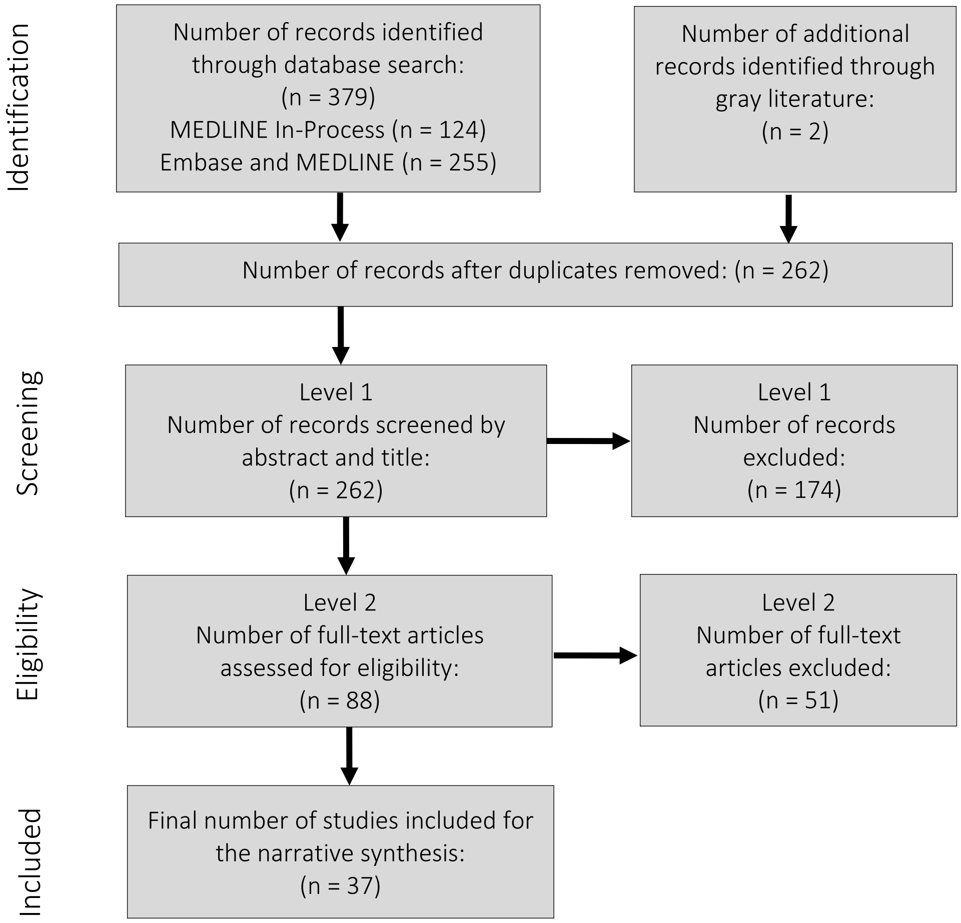

Figure 2. PRISMA flow diagram for the HRQoL SLR

2. Cost and resource use outcomes [*All values in brackets are the inflated values to US$2023]

Table 7. Key findings on the costs associated with chikungunya identified in the SLR on costs

**Study ID**	**Country — year**	**Population**	**Costs reported**	**Direct (estimated) costs**	**Indirect (estimated) costs**	**Total costs (direct + indirect costs/ unspecified)**
Lower- middle income countries						
Hossain et al. (2018)<br>(8)<br><br>Full text	Bangladesh, Dhaka city — 2017	1,326 confirmed and probable CHIKV cases<br>- 1,087 probable cases<br>- 239 confirmed cases	Overall costs during acute chikungunya (consultation, laboratory tests, medicine, transport and special food)	$99.3 [$123.44] overall treatment cost - confirmed cases<br>$26 [$32.32] overall treatment cost - probable cases		
Kaur et al. (2022)<br>(9)<br><br>Full text	India, Gujarat —	60 CHIKV cases	Direct medical costs<br>Non medical costs<br>Indirect costs<br>Illness costs	Rs 5347 [$90.4] total direct medical costs<br>Rs 1865 [$31.54] total non medical costs	Rs 4982 [$84.24] total indirect costs	Rs 15467 [$261.51] total costs due to illness
Vijayakumar et al. (2013)<br>(10)<br><br>Full text	India, Kerala — 2007	1,822 chikungunya patients	Out of pocked expenditure<br>Absenteeism	$2.6 [$3.82] average out of pocket doctor fees<br>$7.5 [$11.02] average out of pocket medicine costs<br>$1.6 [$2.35] average diagnosis costs	$29.4 [$43.21] mean costs due to absenteeism	$15.6 [$22.93] average out-of-pocket health expenditure
Seyler et al. (2010)<br>(11)<br><br>Full text	India, Mallela village — 2005-2006	242 individuals with chikungunya symptoms	Direct costs (medical camp, health centre, hospitalization)	$32.0 [$48.37] direct costs per case<br>$2.10 [$3.17] medical camp costs per patient	$5.5 [$8.31] indirect costs per patient<br>$8.9 [$13.45] productivity loss per patient	$37.5 [$56.68] total costs per patient<br>$9100 [$13,753.91] total costs

			Indirect costs (productivity losses, other indirect costs<br>Total costs	$2.19 [$3.31] health centre costs per patient<br>$6.53 [$9.87] hospitalization per day<br>$7800 [$11,787.07] total direct costs	$1300 [$1,964.84] total in direct costs<br>$2200 [$3,325.12] total productivity losses	
Gopalan et al. (2009)<br>(12)<br><br>Abstract	India, Orissa — 2007	150 primary wage earners from households affected by chikungunya	Out-of-pocket costs<br>Diagnosis costs<br>Loss of income	$84 [$123.44] out-of-pocket costs<br>$77 [$113.16] diagnosis costs	$75 [$110.22] loss of income due to workdays loss (median of 35 days)	
Nandha et al. (2009)<br>(13)<br><br>Abstract	India, Tamil Nadu —	59 individuals with chikungunya symptoms	Treatment costs<br>Loss of income	Rs223 [$7.45] treatment costs per capita	Rs543 [$18.15] average loss of income per capita	
Upper- middle income countries						
Reis et al. (2018)<br>(14)<br><br>Abstract	Brazil — 2016	1,020 beneficiaries from Brazil<br>139 beneficiaries from Rio Grande do Norte (State), Brazil	Total costs			R$1,301,520 [$476,537.49] total cost of chikungunya in Brazil<br>R$163,297 [$59,789.68] total cost of chikungunya in Rio Grande do Norte, Brazil
de Margarette Oliveira de Andrade et al. (2022)	Brazil, Fortaleza — 2017	2683 patients treated as suspected cases of chikungunya at the hospital	Direct costs (hospitalization, emergency care)<br>Absenteeism	$383,514.40 [$476,736.31] total hospital costs	$14,490.90 [$18,013.24] total absenteeism costs of health professionals	

(15)<br><br>Abstract				$174,322.91 [$216,696.06] total emergency care costs<br>$194,700.59 [$242,027] total hospitalization costs		
Claypool et al. (2019)<br>(16)<br><br>Full text	Colombia — 2014–2015	48,321,000 population in Colombia in 2014				$66.3 million [$85.23 million] incremental total costs with insecticide<br>$79.5 million [$102.2 million] incremental total costs with chikungunya vaccine<br>$159.4 million [$204.9 million] incremental total costs with insecticide + chikungunya vaccine
Claypool et al. (2021)<br>(17)<br><br>Full text	Colombia — 2014–2015	48,321,000 population in Colombia in 2014				$15 million [$19.3 million] incremental total costs with insecticide<br>$143 million [$183.8 million] incremental total costs with long-lasting insecticide-treated nets

						$159.4 million [$204.9 million] incremental total costs with insecticide + long-lasting insecticide-treated nets
Alvis-Zakzuk et al. (2018) (18)<br><br>Full text	Colombia — 2014	Chikungunya patients:<br>67 paediatric patients (total)<br>- 58 inpatients<br>- 9 outpatients<br>59 adult patients (total)<br>- 21 inpatients<br>- 38 outpatients	Inpatient costs<br>Outpatient costs<br>Out-of-pocket<br>Indirect costs	$370.4 [$476.74] per paediatric patient (inpatient costs)<br>$318.9 [$410.45] per adult patient (inpatient costs)<br>$93.9 [$120.86] per paediatric patient (outpatient costs)<br>$30.9 [$39.77] per adult patient (outpatient costs)<br>$4.2 [$5.41] per patient (out-of-pocket drug costs)	$0.8 [$1.03] per patient (out-of-pocket transport costs)<br>$81.3 [$104.64] per adult patient (productivity loss)	$152.9 [$196.8] median adult cost per case
Cardona-Ospina et al. (2015)<br>(19)<br><br>Full text	Colombia — 2014	104,141 clinically confirmed cases<br>3,890 laboratory-confirmed cases<br>1,439 both clinically and laboratory-confirmed cases	Management costs			$1,438.74 - $3,396.57 [$1,851.8 - $4,371.71] per patient<br>$73.6 million-$185.5 million [$94.7 million - $238.75 million] outbreak costs

Castañeda-Orjuela et al. (2015)<br>(20)<br><br>Abstract	Colombia — 2014	67 children infected with CHIKV	Inpatient costs	$517.5 [$666.07] per patients ($756.9 [$974.2] in equal or older than 1 year old, and $375.1 [$482.79] in under one year population)		
Vázquez-Cruz et al. (2018)<br>(21)<br><br>Abstract	Mexico, Guerrero — 2015	12,062	Paid sick days		2,397,393.40 pesos [$195,091.71]	
Upper-income countries						
Soumahoro et al. (2011)<br>(22)<br><br>Full text	France, Réunion — 2005-2006	4147 patients included in the estimate of expenses associated with hospitalizations	Direct costs (inpatient visits, consultations, drugs, testing, hospitalization)<br>Indirect costs (productivity losses)	€2,000 [$3,796.53] inpatient costs per patient<br>€26,500,000 [$50,304,296.1] total direct costs<br>€12,400,000 [$23,538,614] total consultation costs<br>€5,000,000 [$9,491,376.6] total drugs costs<br>€570,000 [$1,082,016.9] total serological costs<br>€8,500,000 [$16,135,340.3] total hospitalization costs	€1,360 [$2,581.5] per patient (sick leave)<br>€17,400,000 [$33,029,990] total indirect costs	€43,900,000 [$83,334,286.1] total costs
Feldstein et al. (2019)<br>(23)	U.S., Virgin Islands — 2014	55 estimated inpatient visits (up to 2 months)	Inpatient costs<br>Outpatient costs<br>Absenteeism	$16,982 [$21,857.47] per inpatient visit up to 2 months)	$1,761,000 [$2,266,576.4] (total costs of absenteeism	$33,263,900 [$42,813,838.44] total direct and

Full text		1,295 estimated outpatient visits (up to 2 months)<br>1346 estimated patients (up to 12 months)<br>22775 estimated patients when proportion of population with symptomatic infection = 0.22		$1,526 [$1,964.1] per outpatient visit up to 2 months)<br>$2,915,600 [$3,752,657.6] total direct cost (up to 2 months)<br>$3,536,000 [$4,551,172.1] total direct costs (up to 12 months)	associated with acute and long-term chikungunya up to 12 months)<br>Average cost of absenteeism related to CHIKV:<br>1-2 months: $713-$825 [$917.7 - $1,061.8] ; 6 months: $275-$318 [$353.9 - $409.3]; 12 months: $148-$172 [$190.5 - $221.4].	indirect cost estimate (up to 12 months for estimated patients when proportion of population with symptomatic infection = 0.22)
Abbreviations CHIKV: chikungunya virus; U.S.: United States; Rs: Indian rupee; $: US dollars; R$: Brazilian reais; €: euros.						

Table 8. Key findings on the resource use associated with chikungunya identified in the SLR on resource use

**Study ID**	**Country**	**Population**	**Parameters reported**	**Key findings**
Lower- middle income countries				
Rahim et al. (2018) (24)<br><br>Abstract	Bangladesh, Dhaka — 2017	107 with a confirmed diagnosis of chikungunya	Hospitalization	• Hospitalization rate: 13.1%
Hossain et al. (2018) (8)<br><br>Full text	Bangladesh, Dhaka City — 2017	1,326 confirmed and probable chikungunya cases<br>- 1,087 probable cases<br>- 239 confirmed cases	Absenteeism	• Absenteeism<br>- More than 7 days: approx.. 70% of patients in the acute phase<br>- More than 10 days: 29.6% of patients in the acute phase
Gohel et al. (2019) (25)	India — 2016	110 laboratory-confirmed chikungunya	ICU admission<br>Hospitalization	• Patients with viral encephalitis<br>- ICU admission: 87.5% of patients<br>- Duration of hospital stay: 9.2 days

Full text		cases of which 16 with viral encephalitis		• Patients with viral non-encephalitis<br>- ICU admission: 19.1% of patients<br>- Duration of hospital stay: 5.1 days
Kaur et al. (2022)<br>(9)<br><br>Full text	India, Gujarat	60 chikungunya cases	Absenteeism	• Average absenteeism: 15 days<br>Average absenteeism of family members due to illness: 7 days
Vijayakumar et al. (2013)<br>(10)<br><br>Full text	India, Kerala — 2007	1,822 CHIKV patients	Hospitalization<br>Absenteeism	• Frequency of hospitalization (for at least 1 day): 18.6%<br>Frequency of absenteeism (for at least 1 day): 44.1%
Seyler et al. (2010)<br>(11)<br><br>Full text	India, Mallela — 2005-2006		Hospitalization	• Frequency of medical camp visit: 30% of chikungunya patients<br>• Frequency of health centre visit: 51% of chikungunya patients<br>Mean duration of hospitalization: 5 days
Gupta et al. (2018)<br>(26)<br><br>Full text	India, New Delhi — 2016	60 critically ill patients with CHIKV infection	ICU admission<br>Hospitalization	• Overall<br>- ICU length of stay: 10.23 days<br>- Hospital length of stay: 15.15<br>• Survivors<br>- ICU length of stay: 10.39 days<br>- Hospital length of stay: 15.79<br>• Nonsurvivors<br>- ICU length of stay: 9.95 days<br>- Hospital length of stay: 14.04
Alam et al. (2018)<br>(27)<br><br>Abstract	Pakistan, Karachi	chikungunya patients with persistent arthralgia<br>52	Hospitalization<br>Drug utilization	• Hospitalization rate: 9.6% of patients<br>• Drug utilization:<br>- NSAIDs: 34.6% of patients<br>• - Steroids: 57.7% of patients
Upper-middle income countries				

de Margarette Oliveira de Andrade et al. (2022)<br>(15)<br><br>Abstract	Brazil, Fortaleza — 2017	2683 patients treated as suspected cases of chikungunya at the hospital	Absenteeism	• Duration absenteeism hospital professionals due to CHIKV: 1-9 days<br>• Total absenteeism hospital professionals due to CHIKV: 746 days
Brito Ferreira et al. (2020)<br>(28)<br><br>Full text	Brazil, Recife, Pernambuco — 2014–2016	55 hospitalized laboratory-confirmed CHIKV mono-infection cases and suspected acute neurological disease	Hospitalization<br>Drug utilization	• Duration of hospitalization: 17 days<br>• Drug utilization:<br>- Steroids: 31 patients (61%;)<br>- Immunoglobulin: 12 patients (24%)<br>- Antivirals: 5 patients (9%)<br>- Anticonvulsants: 1 patient (2%)
Chang et al. (2018)<br>(29)<br><br>Full text	Colombia — 2014–2015	485 cases were serologically confirmed with chikungunya	Medication<br>Absenteeism	• Medication:<br>- Acetaminophen: 478 patients (100%)<br>- Ibuprofen: 36 patients (8%)<br>- Prednisone: 5 (1%)<br>- Medicinal Plants: 5 (1%)<br>- Aspirin: 0 patients 0%<br>- Methotrexate: 0 patients 0%<br>Mean absenteeism: 5.5 ± 5.3 days
Hernández et al. (2020)<br>(30)<br><br>Abstract	Colombia — 2014–2018	Chikungunya patients	Hospitalization	• Average yearly hospitalization rate: 9.79%
Castañeda-Orjuela et al. (2015)<br>(20)<br><br>Abstract	Colombia — 2014	Children infected with CHIKV<br>67	Hospitalization<br>Lab requests	• Average length of stay hospitalization: 2.7 days<br>• Lab requests:<br>- Hematic chart: 13.8% of patients<br>- Protein C reactive: 7.57% of patients

Christie et al. (2016)<br>(31)<br><br>Full text	Jamaica — 2014	210 suspected and clinically or laboratory-confirmed CHIKV cases	Hospitalization	• Median duration of hospitalization: 3 days<br>- 0 to 6 months: 4 days<br>- 6.1 months - 6 years: 3 days (n=46)<br>• - 6.1 to 18 years: 2 days (n=28)
Upper income countries				
Marimoutou et al. (2012)<br>(32)<br><br>Full text	France, Reunion —<br>2006–2008	85 self-declared CHIKV+ cases<br>(37 non-healed; 48 healed subjects)	Hospitalization<br>Consultation<br>Absenteeism	• Hospitalization rate: 18% of patients<br>• Number of consultations per patients<br>- Overall health consultations: 9.5 consultations per patients<br>- General practitioner: 5.3 per non-healed patients; 4.1 per healed patient<br>- Functional re-education: 4 per non-healed patient ; 4 per healed patient<br>- Specialist: 0.3 per non-healed patient; 0.02 per healed patient<br>- Acupuncture: 0.6 per non-healed patient; 0.2 per healed patient<br>- Surgery: 0.1 per non-healed patient; 0.1 per healed patient<br>- Emergency: 0.1 per non-healed patient; 0.2 per healed patient<br>• Frequency absenteeism: 62.9% of patients
Marimoutou et al. (2015)<br>(33)<br><br>Full text	France, Reunion —<br>2012	81 self-declared chikungunya+ cases	Consultation<br>Drug utilization	• Frequency of consultations with general practitioner: 93% of patients<br>• Number of consultations:<br>- General practitioner: 6.5 visits per patient<br>- Physiotherapist: 4 visits per patient<br>- Osteopathy: 1.5 visits per patient<br>• Frequency of drug utilization<br>- paracetamol: 77% of patients<br>- nonsteroidal anti-inflammatory: 46% of patients<br>- aspirin: 29% of patients
Soumahoro et al. (2009)<br>(34)	France, Réunion —	199 serology-confirmed CHIKV cases	Hospitalization<br>Drug utilization<br>Consultation	• Frequency of hospitalization in 12 months: 7%<br>• Frequency of analgesic usage: 26%<br>• Frequency of medical consultations: 80%

Full text				
Staikowsky et al. (2008)<br>(35)<br><br>Full text	France, Réunion — 2005	221	Drug utilization<br>Consultations<br>Absenteeism	• Drug utilization:<br>- Paracetamol: 95.4%<br>- Morphine derivatives: 3.7%<br>- Tramadol: 1.4%<br>- Non-steroidal: 55.3%<br>- Corticoids: 27.7%<br>- Quinine + Thiamine: 3.20%<br>- Chloroquine: 1.4%<br>- Hydroxychloroquine: 0.5%<br>- Morinda citrifolia: 12.6%<br>- Cannabis sativa: 10.4%<br>- Eugenia uniflora: 6.3%<br>• Physiotherapy: 13.1%<br>• Absenteeism: 9.9±8.7 days (mean sick leave duration)<br>• Physician consultation: 85.5% of patients
Schilte et al. (2013)<br>(36)<br><br>Full text	France, Reunion — 2006	Total: 180<br>Patients with arthralgia: 62<br>Patients without arthralgia: 33	Hospitalization<br>Consultations<br>Drug utilization	• Frequency of patients with arthralgia among hospitalized patients at 14 months after acute chikungunya: 56.5%<br>• Frequency of patient followed by a GP at 36 months after acute phase:<br>- 80.6% of patients with arthralgia<br>- 3% of patients without arthralgia<br>• Frequency of patient receiving treatment at 36 months after acute phase:<br>- 82.2% of patients with arthralgia<br>- 0% of patients without arthralgia<br>• Frequency of patient receiving continuous treatment at 36 months after acute phase:<br>- 49% of patients with arthralgia<br>- 0% of patients without arthralgia<br>• Frequency of drug utilization:<br>- Paracetamol: 72.5% of patients with arthralgia

				- Non-steroidal anti-inflammatory drugs: 23.5% of patients with arthralgia<br>- Corticosteroids: 5.9% of patients with arthralgia
Basurko et al. (2022)<br>(37)<br><br>Full text	French Guiana	73 pregnant women with CHIKV	Hospitalization	• Hospitalization rate:<br>- Total: 65.7% of cases<br>- Within 24 h: 60% of cases<br>- 24-48 h: 32% of cases<br>• Length of stay between 1-3 days: 75% of cases<br>• ICU rate: 0%
Trentini er al. (2018)<br>(38)<br><br>Full text	Italy — 2007		Hospitalizations	• Length of hospital stay (severe cases): 4.17<br>• Length of hospital stay (mild cases): 3.8<br>• Number of ambulatory visits per symptomatic patient: 2<br>• Hospitalization proportion (severe): 0.48<br>• Hospitalization proportion (mild): 0.001
Pollett et al. (2022)<br>(39)<br><br>Abstract	U.S. – 2014-2018	195 CHIKV cases	Consultations	• Median healthcare visits by individuals with rheumatic CHIKV sequelae: 7 visits
Perti et al. (2016)<br>(40)<br><br>Full text	U.S., Puerto Rico — 2014	180 laboratory-confirmed chikungunya cases from the Veterans Health Administration System<br>- 148 from Puerto Rico<br>- 32 from the USA	Consultations<br>Hospitalization<br>ICU admission	• Frequency of primary care received:<br>- Puerto Rico: 10.8% of patients<br>- USA: 33.3% of patients<br>• Frequency of emergency department care:<br>- Puerto Rico: 86.5% of patients<br>- USA: 66.7% of patients<br>• Frequency of hospitalization:<br>- Puerto Rico: 82 (55.4%)<br>- USA: 6 (20.0%)<br>• Frequency of ICU:<br>- Puerto Rico: 10 (6.8%)<br>- USA: 0 (0%)
Feldstein et al. (2019)	U.S., Virgin		Absenteeism<br>Hospitalization	• Missed work:<br>- 1—2 months: 89% of patients; 5.6 days (mean)

(23)<br><br>Full text	Islands — 2014-2015		Additional healthcare<br>Medication	- 3—6 months: 88% of patients; 2.2 days (mean)<br>- 7—12 months: 9% of patients; 1.2 days (mean)<br>• Missed school:<br>- 1—2 months: 53% of patients; 1.6 days (mean)<br>- 3—6 months: 62% of patients; 3.4 days (mean)<br>- 7—12 months: 7% of patients; 2.1 days (mean)<br>• Hospitalization:<br>- 1—2 months: 9% of patients; 0.4 visits (mean)<br>• Additional healthcare visits:<br>- 1—2 months: 33% of patients; 0.5 visits (mean)<br>- 7—12 months: 25% of patients; 0.6 visits (mean)<br>• Prescribed medication<br>- 7—12 months: 24.19% of patients
Abbreviations: NSAIDs: nonsteroidal anti-inflammatory drugs; ICU: intensive care unit; CHIKV: chikungunya virus; CHIKV+: chikungunya positive; USA: United States of America; GP: general practitioner.				

3. HRQoL results

Table 9. Key findings on the resource use associated with chikungunya identified in the SLR on quality of life

Author	Country	Population	Tool	Key findings
Alam et al. (2018)<br>(41)<br><br>Abstract	Pakistan, Karachi – 2022	112 chikungunya patients with persistent arthritis	VAS (pain)	Treatment with steroids<br>• VAS score >5: 7.57% of patients<br>• VAS score < 5: 72.7% of patients<br>• VAS score 0: 19.7% of patients
Amaral et al. (2019)<br>(42)<br><br>Full text	Brazil – 2018	35 chikungunya patients with arthritis of more than 12 weeks, with painful and swollen joints.	HAQ-DI,<br>VAS (pain)	• HAQ-DI: 1 ±0.40 (mean)<br>• VAS score 7-10: 77% of patients<br>• VAS score 4-6: 20% of patients<br>• VAS score 1-3: 3% of patients<br>• VAS: 8.02 ± 1.82 (mean)
Bosire et al. (2018)<br>(43)<br><br>Abstract	Kenya – 2017-2018	259 acute-febrile participants aged 1 to 17 years	PedsQL	• PedsQL score: 77±0.9

Cardona-Ospina et al. (2015)<br>(19)<br><br>Full text	Colombia – 2014	106,592 chikungunya cases (104,141 clinically confirmed, 3,890 laboratory-confirmed and 1439 cases both clinically and laboratory confirmed)	DALY	Acute disease:<br>• DALYs lost: 1.52 – 1.86 per 100,000 population<br>Chronic disease:<br>• DALYs lost: 39 – 43 per 100,000 population<br>Total:<br>• DALYs lost: 40.44 – 45.14 per 100,000 population
Claypool et al. (2021)<br>(17)<br><br>Full text	Colombia – 2014-2017	Population of Colombia in 2014: 48,321,000	DALYs	DALYs adverted (chikungunya and dengue)<br>Insecticide: 4523 DALYs<br>Long-lasting insecticide-treated nets: 2404 DALYs<br>Insecticide + long-lasting insecticide-treated nets: 6097 DALYs
Couturier et al. (2012)<br>(44)<br><br>Full text	France – 2005-2007	714 patients with a CHIKV infection	AIMS2-SF, GHQ-12, FCI, SF-36	Recovered CHIKV patients:<br>• AIMS2-SF:<br>- Physical: 0.8 ±1.1<br>- Symptom: 1.6 ±1.9<br>- Affect: 2.4 ±1.9<br>- Social interaction: 4.6 ±1.8<br>- Role: 1.0 ±2.1<br>• GHQ-12: 67.1 ±16.0<br>• SF-36:<br>- Physical functioning: 86.4 ±20.7<br>- Role physical: 81.0 ±33.7<br>- Bodily pain: 79.5 ±23.2<br>- Mental health: 67.8 ±18.7<br>- Role emotional: 79.1 ±36.9<br>- Social functioning: 81.8 ±22.5<br>- Vitality: 59.4 ±18.9<br>- General health: 71.1 ±20.4<br>- Physical component summary: 51.8 ±8.3<br>- Mental component summary: 47.2 ±10.6<br><br>Non recovered CHIKV patients:<br>• AIMS2-SF<br>- Physical 1.8 (1.6)

				- Symptom 4.3 (2.5)<br>- Affect 3.6 (2.1)<br>- Social interaction 5.2 (1.6)<br>- Role 2.4 (2.5)<br>• GHQ-12 57.4 (19.8)<br>• SF-36<br>- Physical functioning 67.1 (27.1)<br>- Role physical 46.2 (41.5)<br>- Bodily pain 48.2 (20.8)<br>- Mental health 55.3 (21.5)<br>- Role emotional 50.8 (42.4)<br>- Social functioning 63.1 (25.9)<br>- Vitality 40.1 (19.2)<br>- General health 52.2 (22.9)<br>- Physical component summary 41.2 (10.1)<br>- Mental component summary 40.1 (11.9)<br><br>• FCI score: 1.1 ±1.4
Da Rocha et al. (2017a) (45)<br><br>Abstract	NR	18 patients with chikungunya and neuropathic symptoms	VAS (pain and fatigue)	VAS (pain): 4.4 ±2.4<br>VAS (fatigue): 5.9 ±2.9
Da Rocha et al. (2017b) (45)<br><br>Abstract	Brazil — 2014	Patients with clinical and demographic diagnosis of chikungunya and persistent articular symptoms	HAQ, SF-12, GFS	4–8 weeks of disease:<br>SF-12 — PC: 30.12 ±8.21<br>SF-12 — MC: 40.95 ±12.23<br>HAQ: 1.87 ±0.82<br>GFS: 3.03 ±0.98<br><br>12–16 weeks of disease:<br>SF-12 — PC: 35.86 ±11.11<br>SF-12 — MC: 47.02 ±12.09<br>HAQ: 1.36 ±0.86<br>GFS: 2.53 ±0.95

de Andrade et al. (2010)<br>(46)<br><br>Full text	Réunion, France — 2006	106 CHIKV-infected patients	VAS, SF-MPQ, BPI	BPI: 5.8 ± 2.1<br><br>Patient with pain with neuropathic characteristics<br>Average pain intensity: 6.0 ±1.7<br>SF-MPQ: 15.5 ±5.2<br>SF-MPQ affective: 18.8 ±6.2<br>SF-MPQ sensory 34.3 ±10.7<br><br>Patient with pain with neuropathic characteristics<br>Average pain intensity: 6.1 ± 2.0<br>SF-MPQ: 11.6 ± 5.2<br>SF-MPQ affective: 13.4 ± 6.7<br>SF-MPQ sensory 25.0 ± 9.9
de Oliveira et al. (2019)<br>(47)<br><br>Full text	Brazil — 2017	111 chikungunya patients with chronic musculoskeletal manifestations treated with Pilates	VAS, HAQ, SF-12	Patients with chronic CHIKV<br><br>Pilates group (week 0; week 12)<br>VAS: 6.7 ± 2.4; 4.4 ± 2.4<br>HAQ: 1.7 ± 0.7; 0.7 ± 0.5<br>SF-12—PC: 29.7 ± 8.4; 39.9 ± 9.0<br>SF-12—MC: 41.7 ± 7.3; 47.7 ± 9.7<br><br>Control group<br>VAS: 7.4 ± 2.4; 7.8 ± 2.4<br>HAQ: 1.5 ± 0.6; 1.7 ± 0.6<br>SF-12—PC: 28.7 ± 5.3; 28.9 ± 5.0<br>SF-12—MC: 32.2 ± 7.1; 33.2 ± 7.2
De Souza et al. (2021)<br>(48)<br><br>Full text	Natal, Brazil — 2018	17 individuals with chronic arthralgia due to CHIKV infection	VAS, HAQ, BPI	Active-tDCS (at baseline)<br>VAS: 6,86 ± 1,66<br>HAQ: 1,61 ± 0,42<br>BPI pain severity: 6,0 ± 1,37<br>BPI interference: 7,11 ± 1,47<br><br>Sham-tDCS (at baseline)<br>VAS: 6,07 ± 1,91

				HAQ: 1,14 ± 0,47<br>BPI pain severity: 5,64 ± 1,77<br>BPI interference: 5,63 ± 2,14
Doran et al. (2022) (49)<br><br>Full text	Curaçao, Netherlands – 2017	334 laboratory confirmed chikungunya patients	SF-36	Recovered<br>• SF-36 (2015;2017)<br>- Physical functioning: 84.1 ±24.9; 88.9 ±21.8<br>- Social functioning: 88.0 ±17.3; 95.0 ±11.8<br>- Physical role functioning: 82.5 ±34.9; 87.6 ±30.8<br>- Emotional health perception: 86.0 ±33.3; 94.1 ±22.3<br>- Mental health: 80.7 ±14.8; 89.4 ±13.2<br>- Vitality: 75.6 ±18.4; 85.1 ±15.0<br>- Bodily pain: 80.8 ±21.5; 90.1 ±17.1<br>- General health perception: 74.2 ±15.9; 77.3 ±14.9<br>- Physical component summary: 80.4 ±19.0; 86.0 ±16.6<br>- Mental component summary: 82.6 ±16.7; 90.9 ±12.4<br><br>Mildly affected<br>• SF-36 (2015;2017)<br>- Physical functioning: 72.0 ±25.8; 73.1 ±24.0<br>- Social functioning: 80.9 ±20.7; 83.5 ±20.6<br>- Physical role functioning: 72.7 ±39.4; 69.8 ±43.5<br>- Emotional health perception: 100 ±66.7-100; 80.5 ±37.9<br>- Mental health: 74.5 ±16.5; 82.5 ±16.6<br>- Vitality: 68.3 ±17.2; 72.7 ±18.0<br>- Bodily pain: 72.2 ±23.2; 76.0 ±19.2<br>- General health perception: 63.2 ±18.3; 64.4 ±17.7<br>- Physical component summary: 70.0 ±21.7; 70.8 ±21.1<br>- Mental component summary: 74.6 ±19.9; 79.8 ±19.3<br><br>Highly affected<br>• SF-36 (2015;2017)<br>- Physical functioning: 65.7 ±29.1; 55.3 ±30.9<br>- Social functioning: 77.5 ±23.0; 71.8 ±26.1<br>- Physical role functioning: 62.0 ±44.4; 55.1 ±47.2

				- Emotional health perception: 70.4 ±42.3; 56.8 ±45.6<br>- Mental health: 72.7 ±20.4; 73.0 ±20.3<br>- Vitality: 63.9 ±20.2; 60.9 ±20.7<br>- Bodily pain: 65.1 ±23.2; 58.3 ±22.9<br>- General health perception: 61.1 ±20.2; 55.6 ±23.0<br>- Physical component summary: 63.5 ±24.8; 56.1 ±26.3<br>- Mental component summary: 71.1 ±22.8; 65.6 ±25.0
Doran et al. (2022) (50)<br><br>Full text	Curaçao, Netherlands – 2020	169 chikungunya patients, of which 107 recovered and 62 affected + 151 individuals from the general population (CHIK-)	SF-36; VAS (pain)	Recovered at 60 months after disease onset (n=107)<br>• VAS (pain)<br>- VAS score 0: 73.8%<br>- VAS score mild (1–3): 12.1%<br>- VAS score moderate (4–6): 5.5%<br>- VAS score severe (7–10): 8.4%<br>• SF-36<br>- Physical functioning: 80.4 ±26.0<br>- Social functioning: 90.2 ±16.4<br>- Physical role functioning: 81.3 ±36.0<br>- Emotional health perception: 89.7 ±29.1<br>- Mental health: 85.5 ±15.6<br>- Vitality: 79.7 ±17.3<br>- Bodily pain: 84.7 ±19.7<br>- General health perception: 71.7 ±16.7<br>- Summary physical component: 79.5 ±20.5<br><br>Affected at 60 months after disease onset (n=62)<br>• VAS (pain)<br>- VAS score 0: 73.8%<br>- VAS score mild (1–3): 12.1%<br>- VAS score moderate (4–6): 5.5%<br>- VAS score severe (7–10): 8.4%<br>• SF-36<br>- Physical functioning: 65.6 ±28.4<br>- Social functioning: 84.5 ±17.8<br>- Physical role functioning: 64.5 ±44.0

				- Emotional health perception: 83.3 ±33.5<br>- Mental health: 79.5 ±17.5<br>- Vitality: 67.3 ±20.5<br>- Bodily pain: 65.7 ±20.6<br>- General health perception: 58.3 ±19.3<br>- Summary physical component: 63.5 ±23.7<br><br>CHIK- at 60 months after disease onset (n=151)<br>• SF-36<br>- Physical functioning: 85.9 ±27.0<br>- Social functioning: 92.6 ±16.3<br>- Physical role functioning: 90.1 ±27.9<br>- Emotional health perception: 94.1 ±21.8<br>- Mental health: 87.6 ±15.0<br>- Vitality: 82.1 ±15.1<br>- Bodily pain: 88.5 ±18.4<br>- General health perception: 74.4 ±12.6<br>- Summary physical component: 84.7 ±17.1
Elsinga et al. (2017) (51)<br><br>Full text	Curaçao — 2015	411 participants with a recent acute CHIKV infection	SF-36	Recovered patients<br>• SF-36:<br>- Physical functioning: 88<br>- Role physical: 90<br>- Bodily pain: 87<br>- Mental health: 84<br>- Role emotional: 93<br>- Social functioning: 91<br>- Vitality: 80<br>- General health: 75<br><br>Mildly affected patients<br>• SF-36:<br>- Physical functioning: 79<br>- Role physical: 76<br>- Bodily pain: 75

				- Mental health: 78<br>- Role emotional: 82<br>- Social functioning: 85<br>- Vitality: 71<br>- General health: 69<br><br>Highly affected patients<br>• SF-36:<br>- Physical functioning: 57<br>- Role physical: 56<br>- Bodily pain: 60<br>- Mental health: 67<br>- Role emotional: 61<br>- Social functioning: 73<br>- Vitality: 59<br>- General health: 58
Frye et al. (2018) (52)<br><br>Abstract	Haiti — 2015	171 patients CHIKV symptoms for at least 6 months treated with a single remedy in 12C potency daily for 1 week	VAS, QOL	At month 0<br>VAS (pain): 6.87 ±5.15<br>QOL: 2.27 ±0.62<br><br>At months 7<br>VAS (pain): 4.40 ±8.51<br>QOL: 1.46 ±0.77
Galate et al. (2016) (53)<br><br>Full text	Mumbai, India — 2012-2013	200 patients presenting with acute febrile CHIKV illness	VAS	CHIKV monoinfection<br>VAS score ≤5: 0% of patients<br>VAS score 6-10: 100% of patients<br><br>CHIKV and DENV co-infection<br>VAS score ≤5: 15.78% of patients<br>VAS score 6-10: 84.21% of patients
Gérardin et al. (2018) (54)<br><br>Abstract	Réunion, France — 2015-2016	286 subjects exposed to CHIKV in 2005-2006: 193 infected and 193 non infected	SF-36	Infected individuals:<br>SF-36 physical component: 66.6 ±21.6<br><br>Infected individuals:

				SF-36 physical component:71.0 ± 19.7
Hayd et al. (2020) (55)<br><br>Full text	Roraima, Brazil — 2014	80 participants with a history of CHIKV infection	EQ-5D-5L, MSQ, DAS28	Chikungunya arthritis<br>• DAS-28: 4.36 ± 1.14<br>• EQ-5D:<br>- At least moderate mobility dysfunction: 5/40 (13%)<br>- At least moderate self-care dysfunction: 2/40 (5%)<br>- At least moderate effect on daily activities 4/40 (10%)<br>- At least moderate pain symptoms 23/40 (58%)<br>- At least moderate anxiety and depression symptoms: 14/40 (35%)<br>• MSQ<br>- MSQ stiffness severity: 44%<br>- MSQ physical impact of stiffness: 14.4%<br>- MSQ psychosocial impact of stiffness: 36.9%<br>- Total MSQ scale: 31.8%<br><br>Rheumatoid arthritis controls<br>DAS-28: 5.00 ± 1.53<br>EQ-5D:<br>- At least moderate mobility dysfunction: 14/40 (35%)<br>- At least moderate self-care dysfunction: 7/40 (18%)<br>- At least moderate effect on daily activities: 19/40 (48%)<br>- At least moderate pain symptoms 29/40 (73%)<br>- At least moderate anxiety and depression symptoms: 16/40 (40%)<br>Stiffness measures<br>- MSQ stiffness severity: 45.8%<br>- MSQ physical impact of stiffness: 25.7%<br>- MSQ psychosocial impact of stiffness: 42.2%<br>- Total MSQ scale: 37.9%
Hossain et al. (2018) (8)<br><br>Full text	Dhaka, Bangladesh — 2017	1326 chikungunya patients during acute phase (confirmed and probable cases)	WHOQOL-BREF	WHOQOL-BREF<br>- Environmental health domain: 11.43 ±2.52<br>- Psychological domain: 10.03 ± 2.75<br>- Social relationship domain: 10.02 ±2.94

				- Physical domain: 8.32 ±2.33
Jain et al. (2017) (56)<br><br>Full text	India — 2010-2013	810 CHIKV-infected patients, 572 tested positive by IgM and/ or RT-PCR	VAS	VAS (median): 6.05<br>VAS score 0-5: 34.6% of patients<br>VAS score 6-10: 65.4% of patients
Kamal et al. (2021) (57)<br><br>Full text	Bangladesh — 2019-2020	40 positive CHIKV IgG and/or IgM with VAS score ≥ 4 and DN4 score ≥ 4<br>(intention to treat, n=33) (per protocol, n=21)	VAS, SF-36	VAS (ITT):<br>- amitriptyline group:<br>5.06 ± 0.68 (one week)<br>4.06 ± 0.68 (two weeks)<br>3.18 ± 0.8 (three weeks)<br>2.62 ± 0.71 (four weeks)<br>- duloxetine group:<br>5.23 ± 0.90 (one week)<br>4.35 ± 1.05 (two weeks)<br>3.47 ± 0.87 (three weeks)<br>2.58 ±0.71 (four weeks)<br><br>VAS (PP):<br>- amitriptyline group:<br>5.80 ± 0.78 (one week)<br>5.10 ± 0.56 (two weeks)<br>4.30 ± 0.48 (three weeks)<br>3.40 ± 0.51 (four weeks)<br>- duloxetine group:<br>5.63 ± 1.02 (one week)<br>4.81 ± 0.87 (two weeks)<br>3.81 ± 0.98 (three weeks)<br>2.90 ± 0.70 (four weeks)<br><br>SF-36 (total):<br>- amitriptyline group:<br>61.05 ± 3.10 (four weeks)<br>58.68 ± 5.89 (ten weeks)<br>- duloxetine group:

				61.08 ± 2.42 (four weeks)<br>59.71 ± 4.29 (ten weeks)<br><br>SF-36 (domains):<br>- Amitriptyline group:<br>General health: 50.55 ± 1.66<br>Physical function: 80.11 ± 7.07<br>Limitation of activities: 88.13 ± 11.02<br>Emotional well-being: 62.87 ± 4.47<br>Social activities: 51.11 ± 6.50<br>Bodily pain: 42.60 ± 5.66<br>Vitality: 48.52 ± 2.92<br>Mental health: 55.55 ± 1.66<br><br>- Duloxetine group:<br>General health: 50.55 ± 1.66<br>Physical function: 82.22 ± 6.66<br>Limitation of activities: 89.38 ± 6.25<br>Emotional well-being: 64.88 ± 3.64<br>Social activities: 56.66 ± 8.16<br>Bodily pain: 45.22 ± 5.54<br>Vitality: 50.55 ± 3.03<br>Mental health: 56.11 ± 2.20
Marimoutou et al. (2012)<br>(32)<br><br>Full text	Reunion, France — 2008	85 CHIKV-infected and -297 uninfected individuals. Of the infected 37 are non healed and 48 are healed	SF-36	Non-Healed CHIKV+<br>SF-36 Scale Component<br>- Physical functioning: 83.8 ±16.4<br>- Role, physical: 52.3 ±38.4<br>- Bodily pain: 44.0 ±20.2<br>- General health: 57.8 ±17.7<br>- Vitality: 40.9 ±21.0<br>- Social functioning: 64.5 ±22.9<br>- Role, emotional: 71.2 ±38.6<br>- Mental health: 56.8 ±17.6

				- Physical component summary: 43.6 ±6.8<br>- Mental component summary: 41.6 ±10.8<br><br>Healed CHIKV+<br>SF-36 Scale Component<br>- Physical functioning: 94.5 ±9.5<br>- Role, physical: 85.6 ±28.0<br>- Bodily pain: 65.6 ±26.3<br>- General health: 77.0 ±15.5<br>- Vitality: 61.5 ±15.9<br>- Social functioning: 77.9 ±22.2<br>- Role, emotional: 85.8 ±27.6<br>- Mental health: 69.8 ±13.0<br>- Physical component summary: 52.0 ±6.7<br>- Mental component summary: 47.5 ±7.8<br><br>CHIKV-<br>SF-36 Scale Component<br>- Physical functioning: 94.5 ±9.5<br>- Role, physical: 85.6 ±28.0<br>- Bodily pain: 65.6 ±26.3<br>- General health: 77.0 ±15.5<br>- Vitality: 61.5 ±15.9<br>- Social functioning: 77.9 ±22.2<br>- Role, emotional: 85.8 ±27.6<br>- Mental health: 69.8 ±13.0<br>- Physical component summary: 52.0 ±6.7<br>- Mental component summary: 47.5 ±7.8
Marques et al. (2016) (58)<br><br>Abstract	Brazil — 2016	33 CHIKV infection in patients with and without previous rheumatic disease	VAS	VAS (pain): 6.36 ±2.52<br>VAS (stiffness): 7.44 ±2.11

Martin et al. (2019) (59)<br><br>Abstract	Colombia — NR	548 patients with clinical suspicion of CHIKV infection, 295 were positive for CHIKV IgG and/or CHIKV IgM	HAQ-DI, EQ-5D VAS, VAS for pain	HAQ-DI: 0.17 ±0.45<br>EQ-5D-VAS — VAS: 75.56 ±21.18
Neumann et al. (2021) (60)<br><br>Full text	Brazil — 2017	31 individuals with chronic CHIKV	30-s CST, 40m FPWT, 4SCPT, DASH, VAS, DAS28, SF36, PGIC	Resistance exercise group<br>• Physical function<br>- 40FPWT: W0 42.06±10.6; W6 40.77±11.1; W12 40.23±9.0<br>- 30sCST : W0 8.29±4.1; W6 16.17±8.7; W12 52.79±19.7<br>- 4SCPT: W0 8.78±3.9; W6 14.64±9.1; W12 49.52±20.9<br>- DASH: W0 9.43±4.0; W6 13.54±6.4; W12 51.49±21.0<br>• Pain<br>- VAS: W0 6.43±2.4; W6 4.93±2.6; W12 4.64±2.6<br>- JC: W0 8.50±3.2; W6 8.00±3.9, W12 6.78±4.3<br>• SF-36<br>- Physical functioning: W0 32.50±25.9; W6 31.78±22.6; W12 31.07±24.3<br>- Role physical: W0 12.50±29.0; W6 14.28±18.9; W12 17.86±24.9<br>- Bodily pain: W0 32.64±15.7; W6 37.78±13.5; W12 35.9±18.9<br>- General health: W0 40.00±16.8; W6 38.14±21.3; W12 41.93±18.4<br>- Vitality: W0 36.78±24.5; W6 43.57±22.9; W12 43.57±24.2<br>- Social functioning: W0 52.77±31.5; W6 48.21±28.1; W12 55.35±33.5<br>- Role emotional: W0 35.71±44.3; W6 42.86±47.9; W12 45.24±46.4<br>- Mental health: W0 59.43±22.9; W6 59.71±21.1; W12 57.71±28.9<br><br>Control group<br>• Physical function<br>- 40FPWT: W0 46.09±12.1; W6 46.85±11.5; W12 45.0±11.7<br>- 30sCST: W0 7.06±2.4; W6 6.81±2.2; W12 6.81±1.7

				- 4SCPT: W0 16.01±6.4; W6 16.21±5.6; W12 14.58±6.0<br>- DASH: W0 41.92±12.5; W6 42.65±13.9; W12 45.26±17.0<br>• Pain<br>- VAS: W0 6.12±2.0; W6 6.37±1.7; W12 6.50±1.7<br>- JC: W0 6.62±3.3; W6 6.81±3.2; W12 7.06±3.1<br>• SF-36<br>- Physical functioning: W0 38.44±21.6 ; W6 34.06±16.6 ; W12 36.25±25.8<br>- Role physical: W0 15.62±27.2; W6 12.50±25.8; W12 12.50±25.8<br>- Bodily pain: W0 36.56±19.2; W6 34.06±18.1; W12 37.06±18.4<br>- General health: W0 41.81±16.8; W6 44.00±19.1; W12 47.87±17.9<br>- Vitality: W0 44.12±24.3; W6 40.37±23.2; W12 40.06±22.2<br>- Social functioning: W0 61.72±31.1; W6 53.90±26.5; W12 53.50±20.7<br>- Role emotional: W0 33.3±43.9; W6 31.24±44.6; W12 31.25±43.0<br>- Mental health: W0 53.50±20.7; W6 50.25±20.1; W12 50.25±25.2
Nunes Vidal et al. (2022)<br>(61)<br><br>Full text	Brazil — 2016-2017	236,415 CHIKV cases in 2016<br>and 181,882 in 2017	DALY	• 2016<br>- Total DALYs: 77,422.61<br>- DALY per 1000 inhabitants: 0.3757<br>• 2017<br>- Total DALYs: 59,307.59<br>- DALY per 1000 inhabitants: 0.2856
Padmakumar et al. (2009)<br>(62)<br><br>Full text	India — 2007-2008	120 patients (30 patients per arm) with classical features of chikungunya screened	VAS, ADL, IADL	Aceclofenac alone<br>VAS at visit 0 (cm): 8.30 ± 1.60<br>ADL at visit 0: 10.00 ± 1.17<br>IADL at visit 0: 3.30 ± 0.60<br><br>Aceclofenac plus hydroxychloroquine<br>VAS at visit 0 (cm):7.50 ±<br>ADL at visit 0: 9.67 ± 0.

				IADL at visit 0: 3.17 ± 0.46<br><br>Aceclofenac plus prednisolone<br>VAS at visit 0 (cm): 7.90 ± 1.63<br>ADL at visit 0: 9.93 ± 0.98<br>IADL at visit 03.17 ± 0.46<br><br>Aceclofenac plus hydroxychloroquine plus prednisolone<br>VAS at visit 0 (cm): 7.87 ± 1.46<br>ADL at visit 0: 9.73 ± 0.79<br>IADL at visit 0: 3.23 ± 0.57
Porangaba et al. (2019) (63)<br><br>Abstract	NR — 2018-2019	69 Patients with a diagnosis of chikungunya (confirmed by PCR or serology) with persistent musculoskeletal symptoms after 4 weeks – 58 in followed up in subacute, 50 in chronic 12-24 weeks, and 32 in chronic +24 weeks	VAS, HAQ, SF-12	Subacute phase<br>VAS 6.84±1.9,<br>HAQ 1.59±0.57<br>SF-12 PCS 26.81 ±14.3<br>SF-12 MCS 36.77±15.9.<br><br>Chronic phase (12-24 weeks)<br>VAS 5.27 ± 2.22<br>HAQ 1.16 ± 0.61<br>SF-12 PCS of 38.82±17.26<br>SF-12 MCS 43.72±17.13<br><br>Chronic phase (+24 weeks)<br>VAS 5.64 ± 2.3<br>HAQ 1.11 ± 0.49<br>SF-12 PCS 36.72±19<br>SF-12 MCS 41.56±17.74
Ramachandran et al. (2012)<br>(63)<br><br>Full text	India — 2006	95 non-recovered clinical chikungunya patients, 308 recovered clinical chikungunya patients, 308 healthy individuals	SF-36	Clinically not recovered<br>• SF-36 (median scores)<br>-Physical functioning: 5<br>- Role physical: 0<br>- Body pain: 0<br>- Role emotional: 17

				- Social functioning: 0<br>- Vitality: 19<br>- Mental health: 35<br>- General health: 60<br>Summary Physical: 17<br>Summary Mental: 21<br><br>Clinically recovered<br>• SF-36<br>- Physical functioning: 20ì<br>- Role physical: 25<br>- Body pain: 22<br>- Role emotional: 33<br>- Social functioning: 25<br>- Vitality: 31<br>- Mental health: 40<br>- General health: 62<br>- Summary Physical: 32<br>- Summary Mental: 37<br><br>Healthy normal<br>• SF-36<br>- Physical functioning: 100<br>- Role physical: 100<br>- Body pain: 100<br>- Role emotional: 100<br>- Social functioning: 100<br>- Vitality: 50<br>- Mental health: 40<br>- General health: 85<br>- Summary Physical: 92<br>- Summary Mental: 72
Ravindran et al. (2017) (64)<br><br>Full text	India — 2010	72 adult patients with chronic persistent CA (defined as persistent arthritis for	DAS28 ESR, EULAR, HAQ, VAS	Combination therapy (at fixed dose of methotrexate 15 mg/week, sulfasalazine 1 g daily and HCQ 400 mg daily)<br>DAS28:W0 5.36 ± 0.94, W4 5.00 ± 0.13, W24 3.39 ± 0.87<br>HAQ: 1.94 ± 0.08

		>1 year) after the chikungunya fever		Pain VAS: 64.32 ± 1.75<br><br>Monotherapy with HCQ (dose optimized to 400 mg/day).<br>DAS28: W0 5.36 ± 0.94, W4 4.9 ± 0.69, W24 4.74 ± 0.65<br>HAQ: 1.94 ± 0.08<br>Pain VAS: 64.32 ± 1.75
Rodriguez-Morales et al. (2016)<br>(65)<br><br>Abstract	Colombia — 2010	111 chikungunya patients of which 78 reported post-chikungunya chronic inflammatory rheumatism	SF-36	Post-chikungunya chronic inflammatory rheumatism-negative<br>• SF-36 (median scores)<br>- physical functioning: 85.53%<br>- role physical: 85.53%<br>- bodily pain: 81.62%<br>- vitality: 76.25%<br>- role emotional: 85.96%<br>Post chikungunya chronic inflammatory rheumatism-positive<br>• SF-36 (median scores)<br>- physical functioning: 53.89%<br>- role physical: 41.20%<br>- bodily pain: 51.62%<br>- vitality: 56.42%<br>- role emotional: 54.94%
Rodriguez-Morales et al. (2018)<br>(66)<br><br>Abstract	Colombia — 2015-2017	43 patients with post-chikungunya chronic disease after >2 years (post-chikungunya chronic inflammatory rheumatism-positive) and 19 controls (post chikungunya chronic inflammatory rheumatism-negative).	SF-36, SDS, SAS, PSQI, FSS	Post-chikungunya chronic inflammatory rheumatism-negative<br>• SF-36<br>- physical functioning: 89.5%<br>- role physical: 89.5%<br>- bodily pain: 88.2%<br>- general health: 77.7%<br>- vitality: 79.5%<br>- health transition: 68.4%<br>pCHIK-CD+<br>• SF-36<br>- physical functioning: 62.1%<br>- role physical: 39.0%<br>- bodily pain: 44.4%<br>- general health: 51.4%

				- vitality: 50.6%<br>- health transition: 40.7%
Seyler et al. (2010)<br>(11)<br><br>Full text	India — 2005-2006	242 patients with CHIKV infection symptoms	DALYs	DALYs per case<br>- Acute stage: 0.027<br>- persistent joint pain: 0.011<br>- Total: 0.027<br><br>DALYs Mallela village<br>- Acute stage: 6.50<br>- persistent joint pain: 0.11<br>- Total: 6.60
Simon et al. (2022)<br>(67)<br><br>Full text	Guadeloupe, France — 2013-2015	117 outpatients with suspected chronic chikungunya symptoms	Pain intensity, DN4, SF-12, RAPID-3	Pain intensity (0–10): 6.2 ± 2.4<br>DN4 * (neuropathic pain) (0–10): 4.5 ± 2.3<br>Morning stiffness (0–10): 6.9 ± 2.6<br>Overall stiffness severity (0–10): 7.6 ± 1.7<br>Stiffness impact (0–10): 7.5 ± 2.1<br>Fatigue (0–100): 57.0 ± 24.3<br>SF-12 physical component score: 32.3 ± 7.5<br>SF-12 mental component score: 34.9 ± 9.5<br>RAPID-3 score (0–30): 17.0 ± 7.0
Soumahoro et al. (2009)<br>(34)<br><br>Full text	Reunion — 2007	199 subjects who had serologically confirmed CHIKV infection (CHIKV+) and 199 sero-negative subjects (CHIKV–)	SF-12	CHIKV+ after 1 year from acute infection<br>SF-12—Physical Component: 46.4 ±10.8<br>SF-12—Mental Component: 45.5 ±11.1<br><br>CHIKV-<br>SF-12— Physical Component PC: 49.1 ±9.3<br>SF-12— Mental Component: 45.6 ±10.1
Watson et al. (2019)<br>(68)<br><br>Abstract	Colombia — NR	81 patients with chronic chikungunya	VAS, HAQ-DI, EQ-VAS, MSQ	MSQ: 16%<br>VAS: 65±20<br>HAQ-DI: 0.54±0.52<br>EQ-VAS: 68±62

Watson et al. (2021) (69)<br><br>Full text	Brazil — 2019	40 patients with chronic chikungunya arthritis and 40 CHIKV-negative patients with rheumatoid arthritis (RA)	HAQ, VAS	RA patients<br>Joint pain (VAS): 75 (50–90)<br>HAQ: 0.56 (0.25–1.25)<br><br>CHIKV patients<br>Joint pain (VAS): 73 (50–82)<br>HAQ : 0.50 (0.25–0.75)

Abbreviations: CHIKV: chikungunya virus; chikungunya-: chikungunya negative; chikungunya+: chikungunya positive; QOL: quality of life; GFS: Global Functional Status; PedsQL: pediatric quality of life surveys; FCI: Functional Comorbidity Index; BPI: Brief Pain Inventory; RAPID-3: Routine Assessment of Patient Index Data 3; VAS: visual analogue scale; HAQ: Health Assessment Questionnaire; HAQ-DI: Health Assessment Questionnaire-Disability Index; DALYs: disability-adjusted life year; AIMS2-SF: Arthritis Impact Measurement Scales 2-Short form; GHQ-12: General Health Questionnaire-12 items; SF-36: Short Form Health Survey 36; SF-12: 12-item Short Form Survey; MPQ: McGill Pain Questionnaire; tDCS: transcranial Direct Current Stimulation; EQ-5D-5L: EuroQoL 5-Dimension 5-Level; MSQ: Minnesota Satisfaction Questionnaire; DAS-28: Disease Activity Score 28 for Rheumatoid Arthritis; WHOQOL-BREF: World Health Organization Quality-of-Life Scale; IgG: Immunoglobulin G; IgM: Immunoglobulin M; RT-PCR: Reverse transcription polymerase chain reaction; DN4: Douleur Neuropathique 4 Questions; ITT: intention to treat; PP: per protocol; EQ-5D VAS: EuroQol- 5 Dimension Visual Analogue Scale; 30 30-s CST: Second Sit to Stand Test; 40m FPWT: 40-m Fast-paced Walk Test; 4SCPT: 4-step Stair Climb Power Test; DASH: Disabilities of the Arm, Shoulder, Hand questionnaire; PGIC: Patient Global Impression of Change scale; ADL: Activities of Daily Living; IADL: Instrumental Activities of Daily Living; EULAR: European League Against Rheumatism; SDS: Self-Rating Depression Scale; SAS: Self-Rating Anxiety Scale; PSQI: Pittsburgh Sleep Quality Index; FSS: Fatigue Severity Scale.

4. Risk of Bias

Table 10. Quality assessment of HRQoL studies

**Issues to consider**	**CONCEPTUAL**	**Were the objectives of the study clearly stated?**	**Was a reason provided to justify the HRQoL instrument selected? Was a validated tool used to assess QoL?**	**METHODOLOGY**	**Was the design of the study clearly described? (e.g. cohort, cross-sectional, survey)**	**Was the sampling method for recruitment of participants adequately described?**	**Are inclusion/exclusion criteria clearly described? Do these exclude any individuals that might be relevant?**	**Were characteristics of participants clearly described (demographics and clinical variables)?**	**Was the sample size used appropriately justified?**	**Is it reported who and/or in which clinical setting the instrument was administered?**	**Is the timing of assessments reported? (e.g. baseline and/or at follow-up or after treatment)**	**RESULTS**	**Are response rates reported and if so, are the rates likely to be a threat to validity?**	**Is the loss to follow-up reported and are reasons given? Are these likely to threaten the validity of results**	**Are the levels of missing data reported? How are they dealt with? Could this threaten the validity of results?**	**Were appropriate statistical methods used?**	**INTERPRETATION**	**Were the key findings of the study clearly stated?**	**Were limitations of the study clearly described?**
Alam et al. 2018 (27)		Y	Y		Y	Y	Y	N	Y	N	Y		Y	NA	N	Y		Y	**N**
Amaral et al. 2019 (42)		Y	Y		Y	Y	Y	Y	Y	Y	Y		Y	NA	U	Y		Y	**N**
Bosire et al. 2018 (43)		Y	Y		N	Y	N	N	N	N	N		U	N	N	U		Y	**N**
Cardona-Ospina et al. 2015 (19)		Y	Y		N	NA	NA	Y	Y	NA	NA		NA	NA	NA	Y		Y	**Y**

Claypool et al.<br>2021<br>(17)		Y	Y		Y	Y	Y	Y	Y	Y	NA		NA	Y	NA	Y		Y	**Y**
Couturier et al.<br>2012<br>(44)		Y	Y		Y	Y	Y	Y	Y	N	Y		U	N	N	Y		Y	**Y**
da Rocha et al.<br>2017[a]<br>(45)		Y	Y		N	N	N	N	N	N	N		U	U	U	Y		Y	**N**
da Rocha et al.<br>2017b<br>(45)		Y	Y		Y	N	N	N	N	N	Y		U	U	U	U		Y	**N**
de Andrade et<br>al. 2010<br>(46)		Y	Y		Y	Y	Y	Y	Y	Y	Y		NA	NA	U	Y		Y	**Y**
de Oliveira et<br>al. 2019<br>(47)		Y	Y		Y	Y	Y	Y	Y	Y	Y		Y	Y	U	Y		Y	**Y**
de Souza et al.<br>2021<br>(48)		Y	Y		Y	Y	Y	Y	Y	N	Y		Y	Y	Y	Y		Y	**N**
Doran et al.<br>2022<br>(48)		Y	Y		Y	Y	Y	Y	Y	Y	Y		Y	Y	Y	Y		Y	**Y**
Doran et al.<br>2022<br>(50)		Y	Y		Y	Y	Y	Y	Y	Y	Y		Y	Y	Y	Y		Y	**Y**
Elsinga et al.<br>2017<br>(51)		Y	Y		Y	Y	N	Y	N	Y	Y		Y	N	U	Y		Y	**Y**
Frye et al. 2018<br>(52)		N	Y		N	Y	Y	N	Y	N	Y		Y	U	U	Y		Y	**N**
Galate et al.<br>2016<br>(53)		Y	Y		Y	Y	Y	Y	N	N	N		Y	U	U	NA		Y	**N**
Gerardin et al.<br>2018<br>(54)		Y	Y		N	Y	N	N	N	N	N		U	U	U	U		N	**N**
Hayd et al.<br>2020<br>(55)		Y	Y		Y	Y	Y	Y	N	N	Y		Y	NA	NA	Y		Y	**Y**
Hossain et al.<br>2018<br>(8)		Y	Y		Y	Y	Y	Y	N	N	Y		Y	NA	NA	Y		Y	**Y**
Jain et al. 2017<br>(56)		N	Y		Y	Y	Y	Y	Y	Y	Y		Y	Y	Y	Y		Y	**N**
Kamal et al.<br>2021<br>(57)		Y	Y		Y	Y	Y	Y	Y	U	Y		NA	NA	U	Y		Y	**Y**
Marimoutou et<br>al. 2012<br>(32)		Y	Y		Y	Y	Y	Y	Y	Y	Y		Y	U	U	Y		Y	**Y**
Marques et al.<br>2016<br>(58)		Y	Y		N	N	N	Y	U	N	N		U	U	U	U		Y	**N**

Martin et al. 2019 (59)		Y	Y		N	N	N	N	N	N	N		U	NA	U	Y		Y	**N**
Neumann et al. 2021 (60)		Y	Y		Y	Y	Y	Y	Y	N	Y		Y	U	N	Y		Y	**Y**
Nunes Vidal et al. 2022 (61)		Y	Y		Y	Y	Y	Y	Y	Y	NA		NA	NA	NA	Y		Y	**Y**
Padmakumar et al. 2009 (62)		Y	Y		Y	Y	Y	Y	N	Y	Y		Y	Y	N	Y		Y	**Y**
Porangaba et al. 2019 (70)		Y	Y		N	N	N	Y	N	N	Y		Y	N	U	Y		Y	**N**
Ramachandran et al. 2012 (63)		N	Y		N	Y	Y	Y	N	N	Y		Y	N	U	Y		Y	**Y**
Ravidran et al. 2017 (64)		Y	Y		Y	Y	Y	Y	Y	Y	Y		U	U	U	Y		Y	**Y**
Rodriguez-Morales et al. 2016 (65)		Y	Y		Y	N	Y	N	N	N	N		Y	N	N	U		Y	**N**
Rodriguez-Morales et al. 2018 (66)		N	Y		Y	N	N	N	N	N	N		Y	N	N	U		Y	**N**
Seyler et al. 2010 (11)		Y	Y		N	NA	NA	N	Y	NA	NA		NA	NA	NA	Y		Y	**Y**
Simon et al. 2022 (67)		Y	Y		Y	Y	Y	Y	Y	N	Y		NA	NA	N	Y		Y	**Y**
Soumahoro et al. 2009 (34)		Y	Y		Y	Y	Y	Y	Y	Y	N		Y	N	N	Y		Y	**Y**
Watson et al. 2019 (68)		Y	Y		N	N	N	Y	N	N	N		U	U	U	Y		Y	**N**
Watson et al. 2021 (69)		Y	Y		Y	Y	Y	Y	Y	Y	Y		N	N	N	Y		Y	**Y**
Abbreviations: Y, yes; N, no; NA, not applicable; U, unable to determine																			

Table 11. Quality assessment of cost and resource use studies

**Author and year of publication**	Hernández *et al.*, 2020 (30)	Brito Ferreira *et al.*, 2020 (28)	Gohel *et al.*, 2019 (25)	Rahim *et al.*, 2010 (24)	Alam *et al.*, 2018 (41)	Chang *et al.*, 2018 (29)	Gupta *et al.*, 2018 (29)	Reis *et al.*, 2018 (14)	Castañeda-Orjuela *et al.*, 2015 (20)	Alvis-Zakzuk *et al.*, 2018 (18)	Feldstein *et al.*, 2019 (23)	Cardona-Ospina *et al.*, 2015 (19)	Vijayakumar *et al.*, 2013 (10)	Nandha *et al.*, 2009 (13)	Hossain *et al.*, 2018 (8)

Study design															
**Was the research question stated?**	Y	Y	Y	Y	Y	Y	Y	N	Y	Y	Y	Y	Y	Y	Y
**Was the economic importance of the research question stated?**	Y	N	N	N	N	N	N	N	N	Y	Y	Y	Y	Y	Y
**Was/were the viewpoint(s) of the analysis clearly stated and justified?**	Y	Y	N	N	Y	N	N	N	N	Y	Y	N	N	N	N
**Was a rationale reported for the choice of the alternative programmes or interventions compared?**	N	Y	NA	NA	NA	NA	NA	NA	NA	NA	NA	NA	NA	NA	NA
**Were the alternatives being compared clearly described?**	N	Y	Y	NA	NA	Y	NA	NA	NA	NA	NA	NA	NA	NA	NA
**Was the form of economic evaluation stated?**	NA	NA	NA	NA	NA	NA	NA	NA	Y	Y	Y	Y	Y	N	Y
**Was the choice of form of economic evaluation justified in relation to the questions addressed?**	NA	NA	NA	NA	NA	NA	NA	NA	N	Y	Y	Y	Y	NA	Y
Data collection															
**Was/were the source(s) of effectiveness estimates used stated?**	Y	Y	NA	NA	NA	Y	NA	NA	NA	Y	Y	Y	Y	Y	Y
**Were details of the design and results of the effectiveness study given (if based on a single study)?**	N	NA	NA	NA	NA	NA	NA	NA	NA	Y	NA	NA	Y	NA	NA
**Were details of the methods of synthesis or meta-analysis of estimates given (if based on an overview of a number of effectiveness studies)?**	NA	NA	NA	NA	NA	NA	NA	NA	NA	NA	NA	NA	N	NA	NA
**Were the primary outcome measure(s) for the economic evaluation clearly stated?**	NA	NA	NA	NA	NA	NA	NA	NA	Y	Y	Y	Y	Y	Y	Y
**Were the methods used to value health states and other benefits stated?**	U	Y	Y	Y	Y	Y	Y	NA	NA	Y	Y	Y	NA	Y	Y
**Were the details of the subjects from whom valuations were obtained given?**	NA	Y	Y	Y	Y	Y	Y	N	N	Y	N	N	Y	N	Y
**Were productivity changes (if included) reported separately?**	NA	NA	NA	NA	NA	NA	NA	NA	NA	NA	Y	NA	Y	Y	Y

**Was the relevance of productivity changes to the study question discussed?**	NA	NA	NA	NA	NA	NA	NA	NA	NA	NA	Y	NA	Y	N	Y
**Were quantities of resources reported separately from their unit cost?**	NA	NA	NA	NA	NA	NA	NA	N	U	N	Y	N	N	N	Y
**Were the methods for the estimation of quantities and unit costs described?**	N	NA	NA	NA	NA	NA	NA	N	Y	Y	Y	Y	N	N	N
**Were currency and price data recorded?**	NA	NA	NA	NA	NA	NA	NA	N	Y	Y	Y	Y	Y	N	Y
**Were details of price adjustments for inflation or currency conversion given?**	NA	NA	NA	NA	NA	NA	NA	N	Y	Y	N	N	N	N	N
**Were details of any model used given?**	NA	NA	NA	NA	NA	Y	Y	N	N	NA	NA	NA	NA	N	NA
**Was there a justification for the choice of model used and the key parameters on which it was based?**	NA	NA	NA	NA	NA	NA	Y	N	N	NA	NA	NA	NA	N	NA
Analysis and interpretation of results															
**Was the time horizon of cost and benefits stated?**	NA	NA	NA	NA	NA	NA	NA	NA	N	N	N	N	N	N	N
**Was the discount rate stated?**	NA	NA	NA	NA	NA	NA	NA	NA	N	N	N	N	N	N	N
**Was the choice of rate justified?**	NA	NA	NA	NA	NA	NA	NA	NA	N	N	N	N	NA	N	N
**Was an explanation given if cost or benefits were not discounted?**	NA	NA	NA	NA	NA	NA	NA	NA	N	NA	NA	Y	NA	NA	NA
**Were the details of statistical test(s) and confidence intervals given for stochastic data?**	NA	NA	Y	NA	NA	Y	Y	NA	N	Y	Y	NA	Y	NA	Y
**Was the approach to sensitivity analysis described?**	NA	NA	NA	NA	NA	NA	NA	NA	N	NA	Y	Y	NA	NA	NA
**Was the choice of variables for sensitivity analysis justified?**	NA	NA	NA	NA	NA	NA	NA	NA	N	NA	Y	N	NA	NA	NA
**Were the ranges over which the parameters were varied stated?**	NA	NA	NA	NA	NA	NA	NA	NA	N	Y	Y	N	NA	NA	NA
**Were relevant alternatives compared? (I.e. were appropriate comparisons made when conducting the incremental analysis?)**	NA	NA	Y	NA	NA	Y	Y	NA	NA	NA	Y	NA	NA	NA	NA
**Was an incremental analysis reported?**	NA	NA	NA	NA	NA	N	N	NA	N	N	NA	N	N	NA	N
**Were major outcomes presented in a**	Y	Y	NA	NA	NA	NA	NA	N	N	Y	Y	Y	Y	N	Y

**disaggregated as well as aggregated form?**															
**Was the answer to the study question given?**	Y	Y	Y	Y	Y	Y	Y	NA	Y	Y	Y	Y	Y	Y	Y
**Did conclusions follow from the data reported?**	Y	Y	Y	Y	Y	Y	Y	Y	Y	Y	Y	Y	Y	Y	Y
**Were conclusions accompanied by the appropriate caveats?**	NA	Y	Y	Y	Y	Y	Y	Y	N	Y	Y	Y	Y	Y	Y
**Were the generalizability issues addressed?**	NA	NA	NA	NA	NA	Y	N	NA	N	Y	N	N	N	N	N
**Total Y:**	7	11	9	6	7	12	10	2	8	20	22	15	16	9	17
Author and year of publication	Seyler *et al.*, 2010 (11)	Trentini *et al.*, 2018 (38)	Gopalan *et al.*, 2009 (12)	Perti *et al.*, 2016 (40)	Schilte *et al.*, 2013 (36)	Staikowsky *et al.*, 2008 (35)	Marimoutou *et al.*, 2015 (33)	Soumahoro *et al.*, 2009 (34)	Marimoutou *et al.*, 2012 (32)	Vázquez-Cruz *et al.*, 2018 (21)	Claypool *et al.*, 2021 (17)	Kaur *et al.*, 2022 (9)	de Margarette Oliveira de Andrade *et al.*, 2022 (15)	Claypool et al. 2019 (16)	Basurko et al. 2022 (37)
Study design															
**Was the research question stated?**	Y	Y	Y	U	U	Y	Y	Y	Y	Y	Y	Y	Y	Y	Y
**Was the economic importance of the research question stated?**	Y	Y	Y	N	Y	N	N	Y	N	Y	Y	Y	N	Y	N
**Was/were the viewpoint(s) of the analysis clearly stated and justified?**	N	Y	N	N	N	N	N	N	N	Y	Y	U	U	Y	NA
**Was a rationale reported for the choice of the alternative programmes or interventions compared?**	NA	NA	NA	NA	NA	NA	NA	NA	NA	N	Y	NA	NA	Y	NA
**Were the alternatives being compared clearly described?**	NA	NA	NA	NA	NA	NA	Y	Y	Y	NA	Y	NA	NA	Y	NA
**Was the form of economic evaluation stated?**	Y	Y	Y	NA	N	NA	NA	N	NA	Y	Y	NA	NA	Y	NA
**Was the choice of form of economic evaluation justified in relation to the questions addressed?**	Y	Y	Y	NA	NA	NA	NA	NA	NA	Y	Y	NA	NA	Y	NA
Data collection															
**Was/were the source(s) of effectiveness estimates used stated?**	Y	Y	NA	NA	Y	NA	Y	NA	Y	Y	Y	NA	NA	NA	NA
**Were details of the design and results of the effectiveness study given (if based on a single study)?**	NA	NA	NA	NA	NA	NA	NA	NA	NA	NA	NA	NA	NA	NA	NA

**Were details of the methods of synthesis or meta-analysis of estimates given (if based on an overview of a number of effectiveness studies)?**	NA	NA	NA	NA	NA	NA	NA	NA	NA	NA	NA	NA	NA	NA	NA
**Were the primary outcome measure(s) for the economic evaluation clearly stated?**	Y	Y	Y	NA	Y	NA	NA	NA	NA	Y	Y	Y	Y	NA	Y
**Were the methods used to value health states and other benefits stated?**	Y	NA	NA	Y	Y	Y	Y	Y	Y	NA	Y	NA	NA	Y	NA
**Were the details of the subjects from whom valuations were obtained given?**	N	NA	N	Y	Y	Y	Y	Y	Y	N	Y	Y	Y	NA	Y
**Were productivity changes (if included) reported separately?**	Y	NA	N	NA	NA	NA	NA	NA	Y	Y	NA	NA	Y	NA	NA
**Was the relevance of productivity changes to the study question discussed?**	Y	NA	NA	NA	NA	NA	NA	NA	Y	Y	NA	NA	U	NA	NA
**Were quantities of resources reported separately from their unit cost?**	Y	N	N	NA	NA	NA	NA	NA	NA	Y	NA	NA	Y	NA	Y
**Were the methods for the estimation of quantities and unit costs described?**	Y	Y	N	NA	Y	NA	NA	NA	NA	Y	Y	Y	Y	Y	Y
**Were currency and price data recorded?**	Y	N	N	NA	N	NA	NA	NA	NA	Y	Y	Y	Y	NA	NA
**Were details of price adjustments for inflation or currency conversion given?**	N	N	N	NA	N	NA	NA	NA	NA	N	Y	U	U	NA	NA
**Were details of any model used given?**	Y	Y	N	NA	NA	NA	NA	NA	NA	N	NA	NA	NA	Y	NA
**Was there a justification for the choice of model used and the key parameters on which it was based?**	Y	Y	N	NA	NA	NA	NA	NA	NA	N	Y	NA	NA	Y	NA
Analysis and interpretation of results															
**Was the time horizon of cost and benefits stated?**	N	N	N	NA	NA	NA	NA	NA	NA	Y	Y	NA	NA	Y	NA
**Was the discount rate stated?**	Y	N	N	NA	NA	NA	NA	NA	NA	N	Y	NA	NA	Y	NA
**Was the choice of rate justified?**	N	N	N	NA	NA	NA	NA	NA	NA	N	N	NA	NA	NA	NA
**Was an explanation given if cost or benefits were not discounted?**	NA	NA	NA	NA	NA	NA	NA	NA	NA	NA	N	NA	NA	NA	NA
**Were the details of statistical test(s) and**	N	Y	N	NA	NA	Y	NA	Y	NA	NA	Y	NA	NA	NA	NA

**confidence intervals given for stochastic data?**															
**Was the approach to sensitivity analysis described?**	Y	Y	NA	NA	Y	Y	NA	NA	NA	NA	Y	NA	NA	NA	NA
**Was the choice of variables for sensitivity analysis justified?**	Y	Y	NA	NA	Y	NA	NA	NA	NA	NA	Y	NA	NA	NA	NA
**Were the ranges over which the parameters were varied stated?**	Y	Y	NA	NA	N	NA	NA	NA	NA	NA	Y	NA	NA	NA	NA
**Were relevant alternatives compared? (I.e. were appropriate comparisons made when conducting the incremental analysis?)**	NA	NA	NA	NA	NA	NA	NA	NA	NA	NA	Y	NA	NA	NA	NA
**Was an incremental analysis reported?**	N	N	N	N	N	N	N	N	N	N	U	NA	NA	NA	NA
**Were major outcomes presented in a disaggregated as well as aggregated form?**	Y	N	N	NA	Y	Y	U	Y	Y	N	N	NA	NA	NA	NA
**Was the answer to the study question given?**	Y	Y	Y	Y	Y	Y	Y	Y	Y	Y	Y	Y	Y	Y	Y
**Did conclusions follow from the data reported?**	Y	Y	Y	Y	Y	Y	Y	Y	Y	Y	Y	Y	Y	NA	Y
**Were conclusions accompanied by the appropriate caveats?**	Y	Y	Y	Y	Y	Y	Y	Y	Y	Y	Y	Y	N	NA	NA
**Were the generalizability issues addressed?**	N	Y	N	NA	N	N	N	N	N	N	Y	Y	N	NA	Y
**Total Y:**	22	18	8	5	12	9	8	10	11	16	26	10	9	14	8

References

1. Bramer WM, Rethlefsen ML, Kleijnen J, Franco OH. Optimal database combinations for literature searches in systematic reviews: A prospective exploratory study. Syst Rev. 2017 Dec 6;6(1).
2. Drummond MF, Jefferson TO. Guidelines for authors and peer reviewers of economic submissions to the BMJ. Vol. 313, British Medical Journal. British Medical Journal Publishing Group; 1996. p. 275–83.
3. Mandrik O, Severens J, Bardach A, Ghabri S, Hamel C, Mathes T, et al. Critical Appraisal of Systematic Reviews With Costs and Cost-Effectiveness Outcomes: An ISPOR Good Practices Task Force Report. Value in Health [Internet]. 2021;24:463–72. Available from: https://doi.org/10.1016/j.
4. Papaioannou D, Brazier J, Paisley S. NICE DSU technical support document 9: the identification, review and synthesis of health state utility values from the literature report by the decision support unit [Internet]. 2010. Available from: www.nicedsu.org.uk
5. Soh SE, Morris ME, McGinley JL. Determinants of health-related quality of life in Parkinson's disease: A systematic review. Vol. 17, Parkinsonism and Related Disorders. 2011. p. 1–9.
6. Bartoli S, Aguzzi G, Tarricone R. Impact on Quality of Life of Urinary Incontinence and Overactive Bladder: A Systematic Literature Review. Vol. 75, Urology. 2010. p. 491–500.
7. Picot J, Copley V, Colquitt JL, Kalita N, Hartwell D, Bryant J. The INTRABEAM® photon radiotherapy system for the adjuvant treatment of early breast cancer: A systematic review and economic evaluation. Health Technol Assess (Rockv). 2015 Sep 1;19(69):1–190.
8. Hossain MS, Hasan MM, Islam MS, Islam S, Mozaffor M, Khan MAS, et al. Chikungunya outbreak (2017) in Bangladesh: Clinical profile, economic impact and quality of life during the acute phase of the disease. PLoS Negl Trop Dis. 2018;12(6):1–16.
9. Kaur J, Yadav CP, Chauhan NM, Baharia RK. Economic burden estimation associated with dengue and chikungunya in Gujarat, India. J Family Med Prim Care [Internet]. 2022;11(9):5393–403. Available from: https://journals.lww.com/jfmpc/Fulltext/2022/09000/Economic_burden_estimation_associated_with_dengue.69.aspx
10. Vijayakumar K, George B, Anish TS, Rajasi RS, Teena MJ, Sujina CM. Economic impact of chikungunya epidemic: Out-of-pocket health expenditures during the 2007 outbreak in Kerala, India. Southeast Asian Journal of Tropical Medicine and Public Health. 2013;44(1):54–61.

11. Seyler T, Hutin Y, Ramanchandran V, Ramakrishnan R, Manickam P, Murhekar M. Estimating the burden of disease and the economic cost attributable to chikungunya, Andhra Pradesh, India, 2005-2006. Trans R Soc Trop Med Hyg. 2010;104(2):133–8.
12. Gopalan SS, Das A. Household economic impact of an emerging disease in terms of catastrophic out-of-pocket health care expenditure and loss of productivity: Investigation of an outbreak of chikungunya in Orissa, India. J Vector Borne Dis. 2009;46(1):57–64.
13. Nandha B, Krishnamoorthy K. Cost of illness due to Chikungunya during 2006 outbreak in a rural area in Tamil Nadu. Indian J Public Health. 2009;53(4):209–13.
14. Reis RDU, Neto FAB, Rocha FDD, Miranda RB de, Queiroz MAF de, Moraes CEF, et al. P440 Social Security Impacts of Chikungunya Fever and Rheumatoid Arthritis in 2016 : Analysis of Time Leave from Work in Rio Grande Do Norte and Brazil. Advances in Rheumatology. 2018;(Sbr):21–2.
15. de Margarette Oliveira de Andrade M, de Almeida Barreto FK, Coelho TMS, Praça Pinto G, Timbo Queiroz I, Távora Nogueira C, et al. Chikungunya in Brazil: An epidemic of high cost for private healthcare, 2017. Trop Med Int Health [Internet]. 2022 [cited 2025 Apr 10];(27(10)):925–33. Available from: 10.1111/tmi.13810
16. Claypool AL, Brandeau ML, Goldhaber-Fiebert JD. Quantifying Positive Health Externalities of Disease Control Interventions: Modeling Chikungunya and Dengue. Medical Decision Making. 2019 Nov 1;39(8):1045–58.
17. Claypool AL, Brandeau ML, Goldhaber-Fiebert JD. Prevention and control of dengue and Chikungunya in Colombia: A costeffectiveness analysis. PLoS Negl Trop Dis. 2021 Dec 1;15(12).
18. Alvis-Zakzuk NJ, Díaz-Jiménez D, Castillo-Rodríguez L, Castañeda-Orjuela C, Paternina-Caicedo Á, Pinzón-Redondo H, et al. Economic Costs of Chikungunya Virus in Colombia. Value Health Reg Issues [Internet]. 2018;17(51):32–7. Available from: http://dx.doi.org/10.1016/j.vhri.2018.01.004
19. Cardona-Ospina JA, Villamil-Gómez WE, Jimenez-Canizales CE, Castañeda-Hernández DM, Rodríguez-Morales AJ. Estimating the burden of disease and the economic cost attributable to chikungunya, Colombia, 2014. Trans R Soc Trop Med Hyg. 2015;109(12):793–802.
20. Castañeda-Orjuela C, Díaz-Jiménez D, Rodríguez-Castillo L, Paternina-Caicedo A, Pinzón-Redondo H, Alvis-Guzman N, et al. Medical care costs of Chikungunya Virus infection in a Pediatric Population in Colombia. Value in Health. 2015;18(3):A254–5.
21. Vázquez-Cruz I, Juanico-Morales G, Sánchez-Ramos A, Morales-Sánchez O de J. [Costs and sick leave due to chikungunya in the Instituto Mexicano del Seguro Social in Guerrero, Mexico]. Rev Med Inst Mex Seguro Soc [Internet]. 2018;56(1):54–63. Available from: http://www.ncbi.nlm.nih.gov/pubmed/29368896
22. Soumahoro MK, Boelle PY, Gaüzere BA, Atsou K, Pelat C, Lambert B, et al. The Chikungunya epidemic on La Réunion Island in 2005-2006: A cost-of-illness study. PLoS Negl Trop Dis. 2011;5(6).

23. Feldstein LR, Ellis EM, Rowhani-Rahbar A, Hennessey MJ, Staples JE, Halloran ME, et al. Estimating the cost of illness and burden of disease associated with the 2014–2015 chikungunya outbreak in the U.S. Virgin Islands. PLoS Negl Trop Dis. 2019;13(7):1–14.
24. Rahim M, Zaman S, Mitra P, Jahan I, Chowdhury T, Saha S, et al. Sp210Evaluation of Risk Factors for Acute Kidney Injury Among Patients With Chikungunya: Experience From a Tertiary Care Hospital of a Developing Country. Nephrology Dialysis Transplantation. 2018;33(suppl_1):i414–i414.
25. Gohel S, Modi R, Patel KK, Kumar A, Patel AK. Prevalence, risk factors, and outcome of chikungunya encephalitis in hospitalized patients at a tertiary care center in Gujarat, India, during the 2016 outbreak. Infectious Diseases in Clinical Practice. 2019;27(2):77–80.
26. Gupta A, Juneja D, Singh O, Garg S, Arora V, Deepak D. Clinical profile, intensive care unit course, and outcome of patients admitted in intensive care unit with chikungunya. Indian Journal of Critical Care Medicine. 2018;22(1):5–9.
27. Alam H, Umer TP, Nazir L. SAT0399 Chikungunya fever in karachi: clinical and laboratory features and factors associated with persistent arthralgia. 2018;(June):1062.1-1062.
28. Brito Ferreira ML, Militão de Albuquerque M de FP, de Brito CAA, de Oliveira França RF, Porto Moreira ÁJ, de Morais Machado MÍ, et al. Neurological disease in adults with Zika and chikungunya virus infection in Northeast Brazil: a prospective observational study. Lancet Neurol. 2020;19(10):826–39.
29. Chang AY, Encinales L, Porras A, Reid SP, Martins KAO, Pacheco S, et al. Frequency of Chronic Joint Pain following Chikungunya Infection: A Colombian Cohort Study. Arthritis Rheumatology. 2018;70(4):578–84.
30. Hernández F, Julian G. PIN121 Longitudinal Trends and Burden of Tropical Infectious Diseases in Colombia: A Real-World DATA Analysis. Value in Health [Internet]. 2020;23(December):S564. Available from: https://doi.org/10.1016/j.jval.2020.08.962
31. Christie CDC, Melbourne-Chambers R, Ennevor J, Young-Peart S, Buchanan T, Scott-Brown P, et al. Chikungunya in Jamaica - public health effects and clinical features in children. West Indian Medical Journal. 2016;65(3):431–7.
32. Marimoutou C, Vivier E, Oliver M, Boutin JP, Simon F. Morbidity and impaired quality of life 30 months after chikungunya infection: Comparative cohort of infected and uninfected french military policemen in reunion island. Medicine (United States). 2012;91(4):212–9.
33. Marimoutou C, Ferraro J, Javelle E, Deparis X, Simon F. Chikungunya infection: Self-reported rheumatic morbidity and impaired quality of life persist 6 years later. Clinical Microbiology and Infection [Internet]. 2015;21(7):688–93. Available from: http://dx.doi.org/10.1016/j.cmi.2015.02.024
34. Soumahoro MK, Gérardin P, Boëlle PY, Perrau J, Fianu A, Pouchot J, et al. Impact of Chikungunya virus infection on health status and quality of life: A retrospective cohort study. PLoS One. 2009;4(11):1–6.

35. Staikowsky F, Le Roux K, Schuffenecker I, Laurent P, Grivard P, Develay A, et al. Retrospective survey of Chikungunya disease in Réunion Island hospital staff. Epidemiol Infect. 2008;136(2):196–206.
36. Schilte C, Staikovsky F, Couderc T, Madec Y, Carpentier F, Kassab S, et al. Chikungunya Virus-associated Long-term Arthralgia: A 36-month Prospective Longitudinal Study. PLoS Negl Trop Dis. 2013;7(3):e2137.
37. Basurko C, Hcini N, Demar M, Abboud P, Nacher M, Carles G, et al. Symptomatic Chikungunya Virus Infection and Pregnancy Outcomes: A Nested Case-Control Study in French Guiana. Viruses. 2022;14(12).
38. Trentini F, Poletti P, Baldacchino F, Drago A, Montarsi F, Capelli G, et al. The containment of potential outbreaks triggered by imported Chikungunya cases in Italy: A cost utility epidemiological assessment of vector control measures. Sci Rep. 2018;8(1):1–9.
39. Pollett S, Hsieh HC, Lu D, Grance M, Nowak G, Morales C, et al. The Risk and Risk Factors of Chikungunya Virus Rheumatological Sequelae in Five-Year Virtual Cohort of U.S. Military Health System Beneficiaries. In: Annual Meeting Abstract Book. ASTMH; 2021.
40. Perti T, Lucero-Obusan CA, Schirmer PL, Winters MA, Holodniy M. Chikungunya Fever Cases Identified in the Veterans Health Administration System, 2014. PLoS Negl Trop Dis. 2016;10(5):1–17.
41. Alam H, Perveen T, Nazir L, Khanum I. To Study the Frequency of Persistent Arthritis, in Patients with Chikungunya Fever, in a Tertiary Health Care Center [Internet]. Available from: https://onlinelibrary.wiley.com.
42. Amaral JK, Bilsborrow JB, Schoen RT. Brief report: the disability of chronic chikungunya arthritis. Clin Rheumatol. 2019 Jul 1;38(7):2011–4.
43. Bosire CM, Mutuku F, Krystosik A, Omololu-Aso J, Adams V, Adams D, et al. Chikungunya virus outbreak and malaria co-infection.
44. Couturier E, Guillemin F, Mura M, Léon L, Virion JM, Letort MJ, et al. Impaired quality of life after chikungunya virus infection: A 2-year follow-up study. Rheumatology (United Kingdom). 2012 Jul;51(7):1315–22.
45. da Rocha L, Lima H de, Correia R, Freitas M, Melo P de, Mattos A de, et al. SAT0566 Electroneurographic findings in patients with subacute/chronic articular symptoms of chikungunya fever and neuropathic complaints – preliminary results. In BMJ; 2017. p. 990.3-991.
46. Ciampi de Andrade D, Jean S, Clavelou P, Dallel R, Bouhassira D. Chronic pain associated with the Chikungunya Fever: long lasting burden of an acute illness [Internet]. 2010. Available from: http://www.biomedcentral.com/1471-2334/10/31
47. de Oliveira BFA, Carvalho PRC, de Souza Holanda AS, dos Santos RISB, da Silva FAX, Barros GWP, et al. Pilates method in the treatment of patients with Chikungunya fever: a randomized controlled trial. Clin Rehabil. 2019 Oct 1;33(10):1614–24.

48. De Souza CG, Pegado R, Costa J, Morya E, Baptista AF, Unal G, et al. Alternate sessions of transcranial direct current stimulation (tDCS) reduce chronic pain in women affected by chikungunya. A randomized clinical trial. Brain Stimul. 2021 May 1;14(3):541–8.
49. Doran C, Elsinga J, Fokkema A, Berenschot K, Gerstenbluth I, Duits A, et al. Long-term Chikungunya sequelae and quality of life 2.5 years post-acute disease in a prospective cohort in Curaçao. PLoS Negl Trop Dis. 2022;16(3).
50. Doran C, Gerstenbluth I, Duits A, Lourents N, Halabi Y, Burgerhof J, et al. The clinical manifestation and the influence of age and comorbidities on long-term chikungunya disease and health-related quality of life: a 60-month prospective cohort study in Curaçao. BMC Infect Dis. 2022 Dec 1;22(1).
51. Elsinga J, Gerstenbluth I, Van Der Ploeg S, Halabi Y, Lourents NT, Burgerhof JG, et al. Long-term Chikungunya Sequelae in Curaçao: Burden, determinants, and a novel classification tool. Journal of Infectious Diseases. 2017 Sep 1;216(5):573–81.
52. Frye J, O'keefe J, Fox L, Mannogian H, Legerme M, Prosper J. Individualized Homeopathy Reduces Symptoms of Chronic Chikungunya in Haiti. 2018; Available from: https://www.thieme.de/de/thieme-gruppe/privacy-policy-statement-Thieme-EN.htm
53. Galate LB, Agrawal SR, Shastri JS, Londhey V. Chikungunya Fever Among Patients with Acute Febrile Illness Attending a Tertiary Care Hospital in Mumbai. J Lab Physicians. 2016 Jul;8(02):085–9.
54. Gérardin P, Fianu A, Malvy D, Mussard C, Boussaïd K, Rollot O, et al. Perceived morbidity and community burden after a Chikungunya outbreak: The TELECHIK survey, a population-based cohort study. BMC Med. 2011 Jan 14;9.
55. Luciano R, Hayd N, Maony &, Moreno R, Naveca F, Amdur R, et al. Persistent chikungunya arthritis in Roraima, Brazil. Available from: https://doi.org/10.1007/s10067-020-05011-9
56. Jain J, Nayak K, Tanwar N, Gaind R, Gupta B, Shastri JS, et al. Clinical, Serological, and Virological Analysis of 572 Chikungunya Patients from 2010 to 2013 in India. Clinical Infectious Diseases. 2017 Jul 1;65(1):133–40.
57. Kamal MM, Rahman MM, Sharmin S, Bhowmick DK, Islam MS, Akhtaruzzaman AKM. Efficacy of amitriptyline and duloxetine in post-chikungunya neuropathic pain; A randomized, open-label, cross-over clinical trial. Anaesthesia, Pain and Intensive Care. 2021 Oct 1;25(5):647–52.
58. Marques C, Cavalcanti N, Luna M, Toche P, Andrade C, Dantas A, et al. Chikungunya Fever Outbreak in Brazil: Preliminary Assessment in a Cohort of Patients with Rheumatological Manifestations. 2016.
59. Martin DA, Rueda J, Santos AM, Angarita I, Ballesteros JG, Cuervo F, et al. SAT0458 Evaluation of disability in the Colombia chikungunya epidemic within a colombian copcord study. In BMJ; 2019. p. 1318.2-1319.

60. Neumann IL, de Oliveira DA, de Barros EL, Santos GDS, de Oliveira LS, Duarte AL, et al. Resistance exercises improve physical function in chronic chikungunya fever patients: A randomized controlled trial. Eur J Phys Rehabil Med. 2021 Aug 1;57(4):620–9.
61. Vidal ERN, Frutuoso LCV, Duarte EC, Peixoto HM. Epidemiological burden of Chikungunya fever in Brazil, 2016 and 2017. Tropical Medicine and International Health. 2022 Feb 1;27(2):174–84.
62. Padmakumar B, Jayan JB, Menon RMR, Krishnankutty B, Payippallil R, Nisha RS. Comparative evaluation of four therapeutic regimes in Chikungunya arthritis: A prospective randomized parallel-group study. Indian J Rheumatol. 2009;4(3):94–101.
63. Porangaba M, Siqueira AM, Castelar G. The Impact of Chikungunya Virus Infection on Quality of Life, Functional Status, and Work Ability. Annals of Rhreumatic Diseases. 2019;
64. Ramachandran V, Malaisamy M, Ponnaiah M, Kaliaperuaml K, Vadivoo S, Gupte MD. Impact of Chikungunya on Health Related Quality of Life Chennai, South India. PLoS One. 2012 Dec 12;7(12).
65. Ravindran V, Alias G. Efficacy of combination DMARD therapy vs. hydroxychloroquine monotherapy in chronic persistent chikungunya arthritis: a 24-week randomized controlled open label study. Clin Rheumatol. 2017 Jun 1;36(6):1335–40.
66. Rodriguez-Morales A, Restrepo-Posada V, Acevedo-Escalante N, Rodríguez-Muñoz E, Valencia-Marín M, Castrillón-Spitia J, et al. Impaired Quality Of Life After Chikungunya Virus Infection: A 12-Months Follow-Up Study Of Its Chronic Inflammatory Rheumatism In La Virginia, Risaralda, Colombia. Value in Health. 2016 Nov;19(7):A420–1.
67. Rodriguez-Morales AJ, Gutiérrez-Segura JC, Ocampo-Serna S, Meneses-Quintero OM, Ochoa-Orozco SA, Sánchez-Castaño DM, et al. Post-chikungunya Chronic Disease and Its Impact on Quality of Life, Depression, Anxiety, Fatigue and Sleep Quality: Results From a 2-Year Follow-up Comparative Study of 62 Patients in La Virginia, Risaralda, Colombia. Open Forum Infect Dis. 2018 Nov 26;5(suppl_1):S161–2.
68. Simon F, Bossy R, Federico D, Dezaunay J, Demoux AL, Rugard N, et al. Determinants of Health-Related Quality of Life in Chronic Chikungunya Disease in Guadeloupe. Pathogens. 2022 Sep 1;11(9).
69. Watson H, Tritsch S, Encinales L, Cadena A, Cure C, Porras A, et al. SAT0473 Musculoskeletal stiffness in chikungunya disease: distinct from pain and relevant to quality of life. In BMJ; 2019. p. 1324.3-1325.
70. Watson H, Nogueira-Hayd RL, Rodrigues-Moreno M, Naveca F, Calusi G, Suchowiecki K, et al. Tender and swollen joint counts are poorly associated with disability in chikungunya arthritis compared to rheumatoid arthritis. Sci Rep. 2021 Dec 1;11(1).
